# RBBP4 downregulation increases the sensitivity of A549 and HeLa cells to cisplatin by inhibiting cyclin D1 expression

**DOI:** 10.1016/j.clinsp.2025.100637

**Published:** 2025-04-04

**Authors:** Zhiyu Zeng, Meiqing Mai, Dandan Wang, Jie Ouyang, Zhiru Chen, Jingjing Zhong, Jinjun Rao

**Affiliations:** a Guangdong Provincial Key Laboratory of New Drug Screening, School of Pharmaceutical Science, Southern Medical University, Guangzhou, China; b Department of Pharmacy, Guangxi International Zhuang Medicine Hospital, Nanning, China; c Clinical Pharmacy Center, Nanfang Hospital, Southern Medical University, Guangzhou, China; d Department of pharmacy, Nanfang Hospital, Southern Medical University, Guangzhou, China; e Department of Pharmacy, The Fifth Affiliated Hospital of Jinan University (Heyuan Shenhe people's Hospital), Heyuan, China; f Department of Pharmacy, The Affiliated Guangdong Second Provincial General Hospital of Jinan University, Guangzhou, China; g Department of pharmacy, The People's Hospital of JianYang City, Jianyang, China

**Keywords:** Retinoblastoma-binding protein 4, Cisplatin, Resistance, Cyclin D1, Small interfering RNA

## Abstract

**Introduction:**

Retinoblastoma-Binding Protein 4 (RBBP4), belonging to the WD-40 family, is an important member of the Polycomb Repressor Complex 2 (PRC2), the Nucleosome Remodeling and Deacetylation complex (NuRD), and is involved in chromatin remodeling, histone deacetylation, and H3K27 methylation.

**Methods:**

The effects of cisplatin treatment on cell viability were evaluated using the MTT assay. Western blotting was employed to analyze protein expression, and RNA interference-mediated knockdown of RBBP4 and cyclin D1 was conducted using Lipofectamine 2000. The formation of colonies was evaluated following a 14-day cisplatin treatment period. Cisplatin uptake was quantified by atomic absorption spectrophotometry. RNA sequencing was conducted on total RNA extracted from cells, and lentiviral vectors were employed for gene overexpression, followed by puromycin selection. Immunohistochemistry was performed on tissue microarrays of lung and cervical adenocarcinoma in order to evaluate RBBP4 expression.

**Results:**

In this study, cisplatin was found to induce RBBP4 expression in human lung cancer A549 cells, and RBBP4 expression in cisplatin-resistant A549/DDP cells was significantly higher than in A549 cells. Downregulating RBBP4 expression by small interfering RNA significantly increased the sensitivity of A549 and A549/DDP cells to cisplatin. Conversely, lentiviral-mediated RBBP4 overexpression reduced sensitivity to cisplatin. Mechanistic studies showed that downregulated RBBP4 increased cell sensitivity to cisplatin mainly by inhibiting cyclinD1 expression, and lentiviral-mediated cyclinD1 caused the opposite effects. These same results were verified in human HeLa cells and in cisplatin-resistant HeLa/DDP cells.

**Discussion:**

This study showed that RBBP4 regulates the sensitivity of tumor cells to cisplatin and is a potential target for reversing cisplatin resistance in tumor cells.

## Highlights

• Cisplatin induces RBBP4 in A549 cells, higher in cisplatin-resistant A549/DDP.
• Downregulating RBBP4 with siRNA increased cisplatin sensitivity in A549 and A549/DDP.
• Overexpressing RBBP4 reduced sensitivity, while downregulation inhibited cyclinD1.
• CyclinD1 overexpression reversed this effect in A549, A549/DDP, and HeLa/DDP cells.

## Introduction

Although antitumor-targeted therapy has achieved very encouraging results in recent years, given the limited number of therapeutic targets and rapid development of resistance to targeted drugs, .^1^^,^^2^ clinical applications of this therapy are still restricted, and traditional chemotherapies are still the most important antitumor drugs in clinical practice^3^^,^^4^

Cisplatin has been the most widely used antitumor chemotherapy drug.^5^^,^^6^ However, the resistance of tumor cells to cisplatin is a major problem affecting its clinical use.^7^^,^^8^ Once tumors develop resistance to cisplatin, the therapeutic efficacy of carboplatin is essentially lost and significantly weakened for oxaliplatin.^9^ In view of this, the enhancement of tumor sensitivity to cisplatin is an important goal.

The formation of tumor cell resistance to cisplatin is multifaceted and can be summarized into 4 types as follows:^10^ 1) Pre-target resistance: before platinum binding to DNA, Copper Transporter (CTR1), copper-extruding P-type ATPases (ATP7A and APT7B), Reduced Glutathione (GST), Multidrug Resistance Protein-2 (MRP2), and other major related factors affect the transmembrane transport and activity of platinum.^11^^,^^12^ 2) On-target resistance: related factors that affect DNA repair and formation of drug resistance at multiple stages include Excision Repair Cross-Complementing rodent repair deficiency complementation group 1 (ERCC1), Breast Cancer 1/2 (BRCA1/BRCA2), Voltage-Dependent Anion Channel (VDAC), Polymerase Eta (POLH), and catalytic (REV3) and structural (REV7) subunits of the translesion synthesis DNA polymerase.^13^^,^^14^ 3) Post-target resistance: major related factors that induce changes in cell survival and apoptosis signaling pathways include cyclinD1, .^15^^,^^16^ BCL-2-like proteins, caspases, TP53, and surviving.^17^^,^^18^ 4) Off-target resistance: activation of some compensatory mechanisms in cells such as autophagy, Heat-Shock Proteins (HSPs), and dual-specificity Y-phosphorylation-regulated kinase 1B (DYRK1B) .^19^^,^^20^ Research on cisplatin resistance has been a popular topic for a long time, but the above problems have not been solved effectively.

Retinoblastoma Binding Protein 4 (RBBP4, also known as RbAP48 and NURF55) is a nuclear protein with a molecular weight of 48 kDa that belongs to the WD family of proteins.^21^ It is involved in the composition of a variety of protein complexes and is an important member of the Nucleosome Remodeling and Deacetylation complex (NuRD), Polycomb Repressor Complex 2 (PRC2) .^22^, ^23^ It participates in chromatin remodeling, histone deacetylation, and H3K27 methylation and in regulating histone acetylation and methylation levels, cell differentiation, development, and self-renewal in stem cells.^24^^,^^25^ It is a key factor in maintaining cell stability and development. Kitange et al. found that RBBP4 downregulation increases cerebral glioblastoma sensitivity to temozolomide by inhibiting O^6^-methylguanine-DNA Methyltransferase (MGMT), RAD51, and Fidgetin Like-1 (FIGNL1) expression.^26^ The previous study has shown that downregulated RBBP4 expression inhibits Epithelial Mesenchymal Transition (EMT) in MS751 human cervical cancer cells.^27^ Therefore, the authors speculated that RBBP4 may affect the sensitivity of tumor cells to cisplatin.

In the present study, the authors found that RBBP4 was overexpressed in human cisplatin-resistant A549/DDP lung adenocarcinoma epithelial and HeLa/DDP cervical cancer cell lines. Small interfering RNA (siRNA) downregulated RBBP4 and subsequently inhibited cyclinD1 expression, thereby enhancing cisplatin sensitivity in these tumor cells. These findings suggest that RBBP4 is a potential target for reversing tumor cell resistance to cisplatin.

## Materials and methods

### Cell culture

The human lung adenocarcinoma epithelial cell line, A549, and the cervical cancer cell line, HeLa, were purchased from the China Center for Type Culture Collection in Shanghai. The cisplatin-resistant subline A549/DDP was kindly provided by Prof. GuiJun Huang (Dept. of Respiratory Illness, Xinqiao Hospital, the Third Military Medical University, Chongqing, China). The cisplatin-resistant subline HeLa/DDP was kindly provided by Prof. Le Yu (School of Pharmaceutical Sciences, Southern Medical University, Guangzhou, China). The A549/DDP cell line was developed from human lung adenocarcinoma A549 cells through long-term exposure to increasing cisplatin concentrations, resulting in cisplatin resistance. These cells exhibit epithelial-like morphology and grow adherently. Similarly, the HeLa/DDP cell line was generated via cisplatin induction. The A549/DDP cell line has an epithelial-like morphology and grows adherently to the wall. Similarly, the HeLa/DDP cell line was obtained by cisplatin induction. Cells were cultured in RPMI-1640 medium (Gibco, CA, USA) supplemented with 10 % fetal bovine serum at 37 °C in a humidified atmosphere of 5 % CO_2_. All cell lines are routinely subjected to STR profiling.

### Cisplatin treatment and cell viability assay

Cells were seeded onto 96-well plates at a density of 3 × 10^3^/well. After 24 h, cells were treated with varying doses of cisplatin (Sigma-Aldrich, St. Louis, MO, USA) for 48h Subsequently, 10 μL 3-(4,5 dimethylthiazol-2-yl)-2,5-diphenyl-tetrazoliumbromide (MTT, 5 mg/mL) were added and incubated with the cells at 37 °C for 4h The absorbance was measured at 570 nm using a microplate reader (Bio-Rad Laboratories, Hercules, CA, USA).

### Western blotting analysis

Cells were collected, and protein extracts were obtained by incubating cells with RIPA Lysis Buffer (Beyotime, Shanghai, China) on ice for 25 min, after which cell lysates were centrifuged for 30 min at 12,000 rpm, and the clear supernatant was collected. Protein concentrations in the supernatants were determined using the BCA Protein Assay Kit (Thermo Fisher Scientific, Waltham, MA, USA). Cell lysates (40 μg) were separated by 12 % SDS–PAGE (Bio-Rad) and transferred to nitrocellulose membranes (Pall Corporation, NY, USA). After blocking with 5 % fat-free dry milk in TBST (50 mM Tris–HCl, 150 mM NaCl, pH 7.4, 0.1 % Tween-20) for 1 h at room temperature, the membranes were independently incubated with specific primary antibodies (1:2000 anti-RBBP4 antibodies, Abcam, Cambridge, UK; 1:1000 cyclinD1 and β-actin, Cell Signaling Technology, Beverly, MA, USA) overnight at 4 °C. Subsequently, the membranes were incubated with Horseradish Peroxidase (HRP)-conjugated anti-rabbit secondary antibody (1:4000 dilution, Cell Signaling Technology) at room temperature for 1h Protein brands were visualized using an ECL detection system (Tanon, Shanghai, China).

### RNA interference

siRNAs were used to silence RBBP4 (siRBBP4, sense: 5′CCAGΜGGCΜΜCCAGAΜGΜA dTdT3’, and antisense: 3′dTdT GGΜCACCGAAGGΜCΜACAΜ 5′), cyclinD1 (siCyclinD1, sense: 5′GCAUGUUCGUGGCCUCUAA dTdT3’, and antisense: 3′dTdT CGUACAAGCACCGGAGAUU 5′), or were Negative Control siRNAs (NC) (designed and synthesized by Ribobio Co. Ltd., Guangzhou, China). Cells were seeded and grown to 40 %–50 % confluence in growth medium. The transfection was carried out using Lipofectamine 2000 (Invitrogen, Carlsbad, CA, USA) in accordance with the manufacturer's instructions.

### Colony formation assay

After a 12h transfection, cells in the blank, negative control, and siRBBP4 groups were digested with 0.25 % trypsin, and then 200 cells were inoculated into 60 mm diameter culture dishes and incubated for 24 h, followed by incubating the cells with cisplatin at the defined concentrations in a 37 °C incubator containing 5 % CO_2_ for 14 days. After 3 washes in PBS, the cells were fixed in absolute ethanol and stained with crystal violet for colony counting.

### Determination of cisplatin by atomic absorption spectrophotometry

Cells cultured in 100 mm culture dishes were treated with siRBBP4 for 48 h, followed by washing in PBS twice, then culture medium containing cisplatin was added at the defined concentrations and cultured at 37 °C in an incubator containing 5 % CO_2_ for 2h The cells were digested with 0.25 % trypsin and washed in PBS 3 times before being resuspended in 1 mL of deionized water. Two hundred microliters of cell suspension were used to determine the protein content using the Bradford method. The remaining 800 μL of cell suspension was added into an Eppendorf tube and centrifuged to remove the supernatant. After vacuum drying, the cell pellet was resuspended in 200 μL 70 % nitric acid and reacted at 70 °C for 2h An atomic spectrophotometer (Hitachi, Z-2000) was used to determine the platinum content in the cell suspension.

### RNA sequencing

RNA sequencing was carried out by RiboBio Co., Ltd. The main steps were as follows: total RNA was isolated from cells using the Trizol reagent (Invitrogen) according to the manufacturer's protocol. RNA purity was assessed using a ND-1000 Nanodrop. Each RNA sample had an A260:A280 ratio of approximately 1.8 and A260:A230 ratio of approximately 2.0. RNA integrity was evaluated using an Agilent 2200 TapeStation (Agilent Technologies, Santa Clara, CA, USA), and each sample had an RINe above 7.0. Briefly, RNAs were ligated with a 3′RNA adapter followed by 5′ adapter ligation. Subsequently, the adapter-ligated RNAs were subjected to RT-PCR and amplified with a low-cycle number. Then the PCR products were size selected by PAGE according to the instructions of the NEBNext® Multiplex Small RNA Library Prep Set for Illumina® (NEB, Ipswich, MA, USA). The purified library products were evaluated using the Agilent 2200 TapeStation and diluted to 2 pM for cluster generation in situ on the HiSeq2500 single-end flow cell followed by sequencing (1 × 50 bp) on HiSeq2500.

### Lentiviral mediated gene expression

The lentiviral expression vectors were constructed by Obio Technology Co. Ltd. (Shanghai, China). The vector carrying the RBBP4 gene (H4061) was pLOV-EF1a-PuroR-CMV-EGFP-2A-RBBP4–3Flag, and the control vector (CN179) was pLOV-EF1a-PuroR-CMV-EGFP-2A-3Flag. The vector carrying the cyclinD1 gene (H6102) was pLenti-EF1a-EGFP-P2A-Puro-CMV-cyclinD1–3Flag, and the control vector (H149) was pLenti-EF1a-EGFP-P2A-Puro-CMV-3Flag.

Cells in the logarithmic growth phase were trypsinized to prepare single cell suspensions followed by seeding at a density of 1.5 × 10^5^ cells per well in 6 well culture plates. After 24 h incubation, cells were transfected with lentivirus at the optimal Multiplicity of Infection (MOI) of 10 pfu/μL with 5 μg/mL final concentration of polybrene. Cells were incubated in a 37 °C incubator containing 5 % CO_2_ for 20h The old medium was aspirated from the culture followed by adding 2 mL fresh culture medium for a 72 h culture, and the efficiency of transfection was observed by a fluorescence microscope. The culture medium was changed to a complete culture medium containing 2 μg/mL puromycin to continue culturing the cells for 2 weeks, followed by detecting the protein expression by fluorescence microscopy and western blotting.

### Tissue microarrays and immunohistochemistry (IHC) analysis

This study was approved by the Ethics Committee of Shanghai Outdo Biotech Company (No. YB M-05–02). The Tissue Microarray (TMA) of lung adenocarcinoma (HLug-Ade150Sur-01, Shanghai Outdo Biotech Co. LTD, Shanghai, China) consisted of 75 paired lung adenocarcinoma and adjacent normal samples, and the TMA of cervical adenocarcinoma (Xi'an Elena Biotechnology Co. LTD, Xi'an, China) consisted of 11 cervical adenocarcinomas and 16 adjacent normal samples. Human Protein Atlas (HPA) database (https://www.proteinatlas.org/) was used for normal tissue (Patient ID: 2101 and 1895). For immunohistochemical staining, first, a section of tissue sample was baked at 63 °C for 1 h, and then the section was deparaffinized with xylene and rehydrated. After that, antigen retrieval was carried out by cooking sections in boiled citric acid solution (pH = 6.0) for 5 min. Then, endogenous peroxidase activity was blocked with a Peroxidase-Blocking Reagent (38.4 mL anhydrous methanol, 12 mL 30 % H_2_O_2,_ 9.6 mL ddH_2_O) for 15 min at room temperature, followed by sequential incubations with rabbit anti-RBBP4 antibody (1:400, Abcam), secondary antibody (Envision+/HRP, Rabbit, DAKO), and finally development with Diaminobenzidine Tetrahydrochloride (DAB).

All of the staining was assessed by two independent pathologists blinded to the sample origin and subject outcome, based on both the proportion of positively stained tumor cells and the intensity of staining. The staining percentage was divided into: 1) Stained tumor cells ≤ 30 %; 2) Stained tumor cells = 30 %–70 %; 3) Stained tumor cells ≥ 70 %. The staining intensity was scored as: 1) Negative staining or weak staining; 2) Moderate staining; 3) Strong staining. This study followed the STROBE guideline.

### Statistical analysis

Data are shown as means ± Standard Deviation (SD), and Student's *t*-tests (unpaired, two-tailed) were conducted using Prism GraphPad software (San Diego, CA, USA); p-values < 0.05 were considered statistically significant. CalcuSyn2.0 software was used to calculate the Combination Index (CI). A CI value less than 1 indicated that the two drugs had a synergistic effect; greater than 1 indicated antagonism; and equal to 1 indicated a nearly additive effect.

## Results

### Cisplatin-induced RBBP4 expression and was elevated in cisplatin-resistant A549/DDP cells

**Fig. 1:**
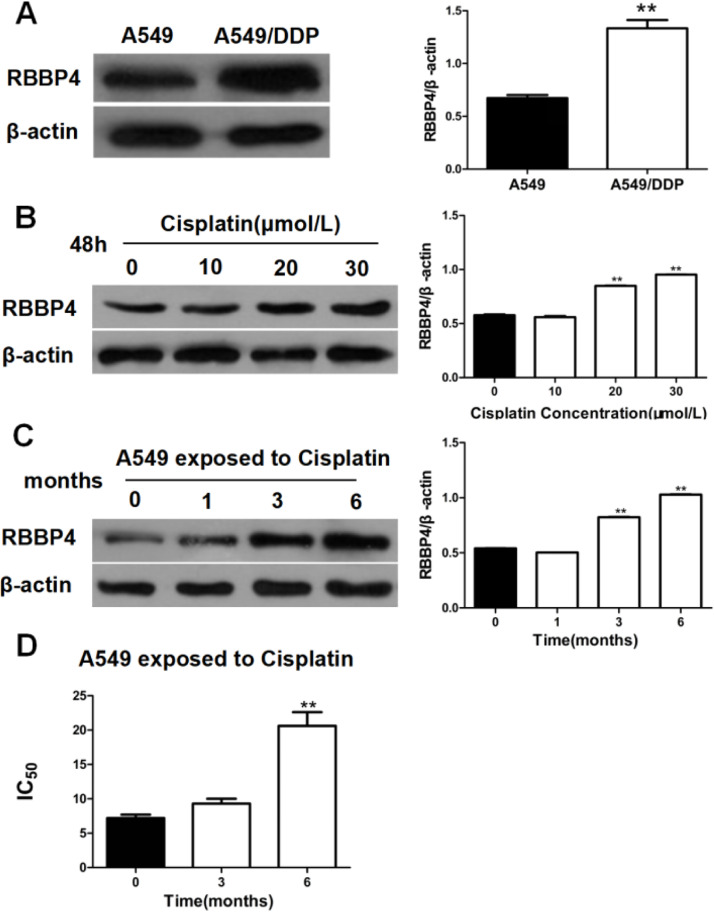
**Cisplatin-induced RBBP4 expression and was elevated in cisplatin-resistant A549/DDP Cells.** (A) RBBP4 expression in cisplatin-resistant A549/DDP cells was significantly higher than in A549 cells. (B) A549/DDP cells treated with cisplatin for 48 h had significantly higher RBBP4 expression. (C) RBBP4 expression in cells treated with cisplatin gradually increased. (D) The IC_50_ of A549 cells to cisplatin was gradually increased. Data are reported as means ± SD with three replicates. *p < 0.05, **p < 0.01, ***p < 0.001. RBBP4, Retinoblastoma-Binding Protein-4.

Western blotting analysis revealed significantly higher RBBP4 expression in cisplatin-resistant A549/DDP cells (IC_50_ =38.10±4.33 μM) compared to non-resistant A549 cells (IC_50_=7.20±0.87 μM). Dose-dependent increases in RBBP4 expression were observed when A549/DDP cells were treated with different concentrations of cisplatin for 48 (Fig. 1A‒B). In a long-term treatment experiment, A549 cells exposed to progressively higher cisplatin concentrations (up to 6 μmoL/L over six months) showed gradual increases in RBBP4 expression (Fig. 1C) and IC_50_ to cisplatin (20.62±3.45 μmoL/L; Fig. 1D). These findings suggest that cisplatin exposure induces RBBP4 expression, contributing to acquired resistance.

### Downregulation of RBBP4 enhanced cisplatin sensitivity in A549 and A549/DDP cells

**Fig. 2:**
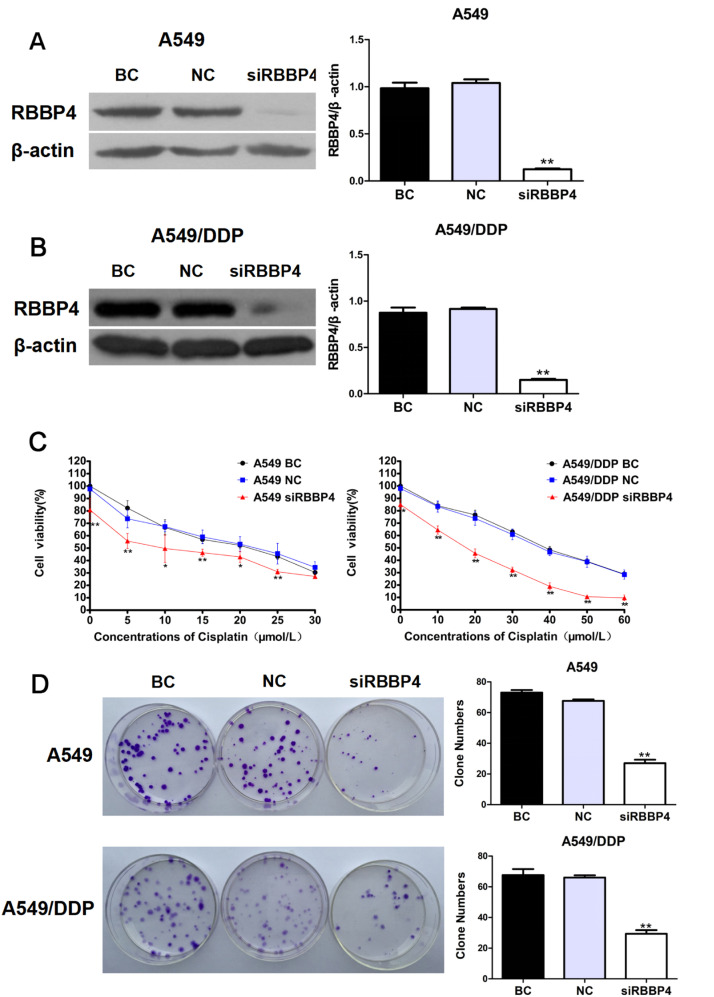
**Downregulation of RBBP4 enhanced cisplatin sensitivity in A549 and A549/DDP cells.** (A) siRNA-induced RBBP4 downregulation affected the cisplatin sensitivity in A549 cells. (B) siRNA-induced RBBP4 downregulation affected the cisplatin sensitivity in A549/DDP cells. (C) MTT showed that RBBP4 downregulation enhanced the cisplatin sensitivity in A549 and A549/DDP cells. (D) Colony formation assays showed that RBBP4 downregulation enhanced the cisplatin sensitivity in A549 and A549/DDP cells. Data are reported as means ± SD with three replicates. *p < 0.05, **p < 0.01, ***p < 0.001. RBBP4, Retinoblastoma-Binding protein-4; BC, Blank Control; NC, siRBBP4 Negative Control.

Silencing RBBP4 with siRNA significantly increased cisplatin sensitivity in both A549 and A549/DDP cells, as confirmed by MTT and colony formation assays (Fig. 2A‒D). Additionally, RBBP4 knockdown alone inhibited cell growth. Combination Index (CI) analysis using CalcuSyn software indicated that combining siRBBP4 and cisplatin treatment had a synergistic effect on inhibiting tumor cell growth, with CI values ranging from 0.332‒0.860 for A549 cells and 0.303‒0.972 for A549/DDP cells. These results demonstrate that RBBP4 downregulation enhances the therapeutic efficacy of cisplatin.

### RBBP4 overexpression reduced cisplatin sensitivity in A549 cells

**Fig. 3:**
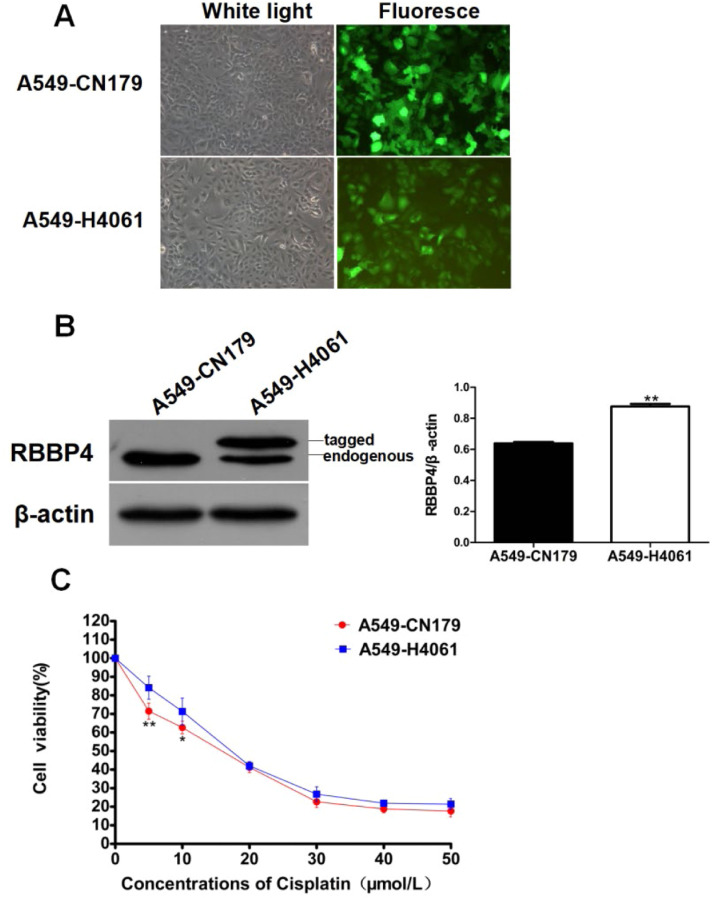
**RBBP4 overexpression reduced cisplatin sensitivity in A549 cells.** (A‒B) A549-H4061 cells expressed both exogenous and endogenous RBBP4 protein. (C) The sensitivity of A549-H4061 cells to cisplatin was lower than A549-CN179 cells. Data are reported as means ± SD with three replicates. *p < 0.05, **p < 0.01, ***p < 0.001. RBBP4, Retinoblastoma-Binding Protein-4.

To further investigate RBBP4′s role, the authors established an RBBP4-overexpressing A549 cell line (A549-H4061) using lentiviral transduction. A549-H4061 cells exhibited higher RBBP4 levels than control cells (Fig. 3A‒B) and were less sensitive to cisplatin at 5‒10 μmoL/L (Fig. 3C). However, the effect diminished at cisplatin concentrations >10 μmoL/L, suggesting that RBBP4 overexpression alone is insufficient to confer high cisplatin tolerance. This indicates that cisplatin resistance involves multiple factors beyond RBBP4 expression.

### RBBP4 downregulation suppressed cyclin D1 expression in A549 cells

**Fig. 4:**
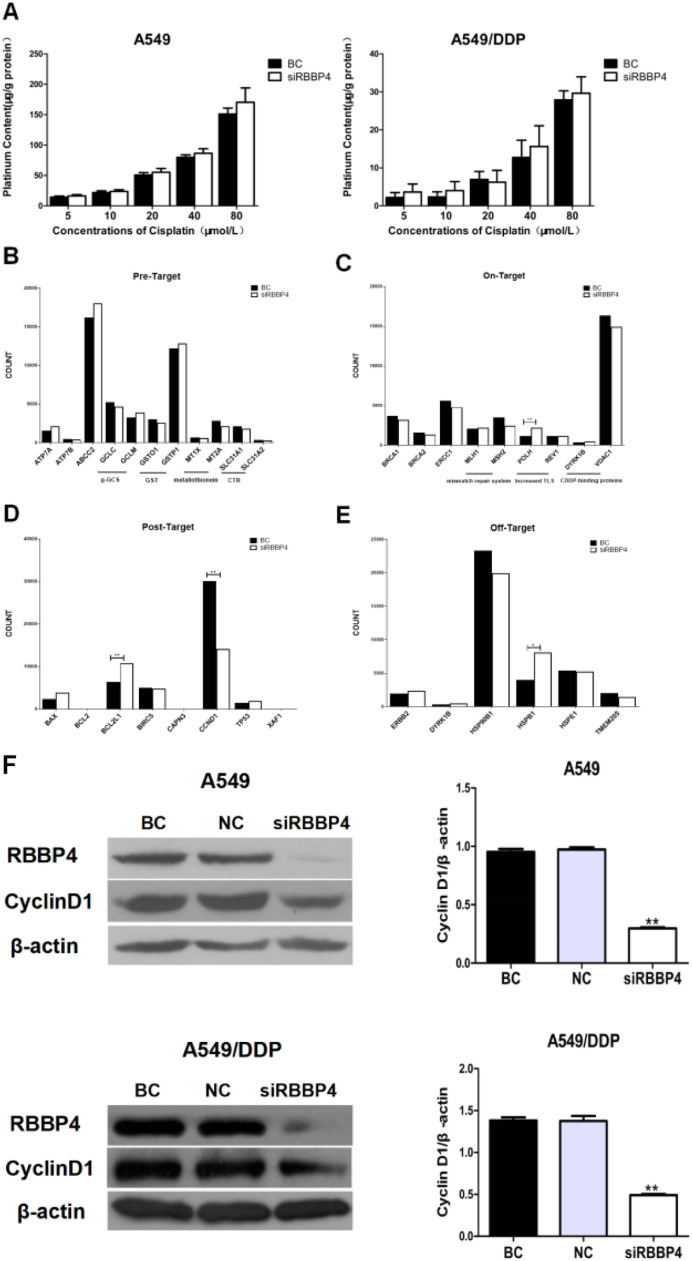
**RBBP4 downregulation suppressed Cyclin D1 expression in A549 cells.** (A) RBBP4 downregulation resulted in no significant change in intracellular platinum content in A549 and A549/DDP cells. (B‒E) The vast majority of known cisplatin-resistance-related genes had no changes; *POLH, Bcl2L1*, and *Hspb1* expressions were increased, and *cyclinD1* expression was suppressed. (F) RBBP4 downregulation inhibited cyclinD1 expression in A549 and A549/DDP cells. Data are reported as means ± SD with three replicates. *p < 0.05, **p < 0.01, ***p < 0.001. RBBP4, Retinoblastoma-Binding Protein-4; BC, Blank Control; NC, siRBBP4 Negative Control.

To explore the mechanism underlying RBBP4-mediated cisplatin resistance, the authors first analyzed intracellular platinum content, which was unaffected by RBBP4 downregulation (Fig. 4A). RNA sequencing identified significant changes in 468 genes after RBBP4 knockdown, including 303 upregulated and 165 downregulated genes (Supplementary Tables S1 and S2). Notably, most cisplatin-resistance-related genes showed no significant changes except for increased expression of POLH, Bcl2L1, and Hspb1, and suppressed expression of cyclin D1 (Fig. 4B‒E). Since POLH, Bcl2L1, and Hspb1 promote resistance, their upregulation is unlikely responsible for the enhanced sensitivity. Western blot confirmed that RBBP4 knockdown significantly reduced cyclin D1 expression (Fig. 4F), suggesting cyclin D1 suppression as a key mechanism by which RBBP4 downregulation enhances cisplatin sensitivity.

### Cyclin D1 suppression was crucial for cisplatin sensitization by RBBP4 downregulation

**Fig. 5:**
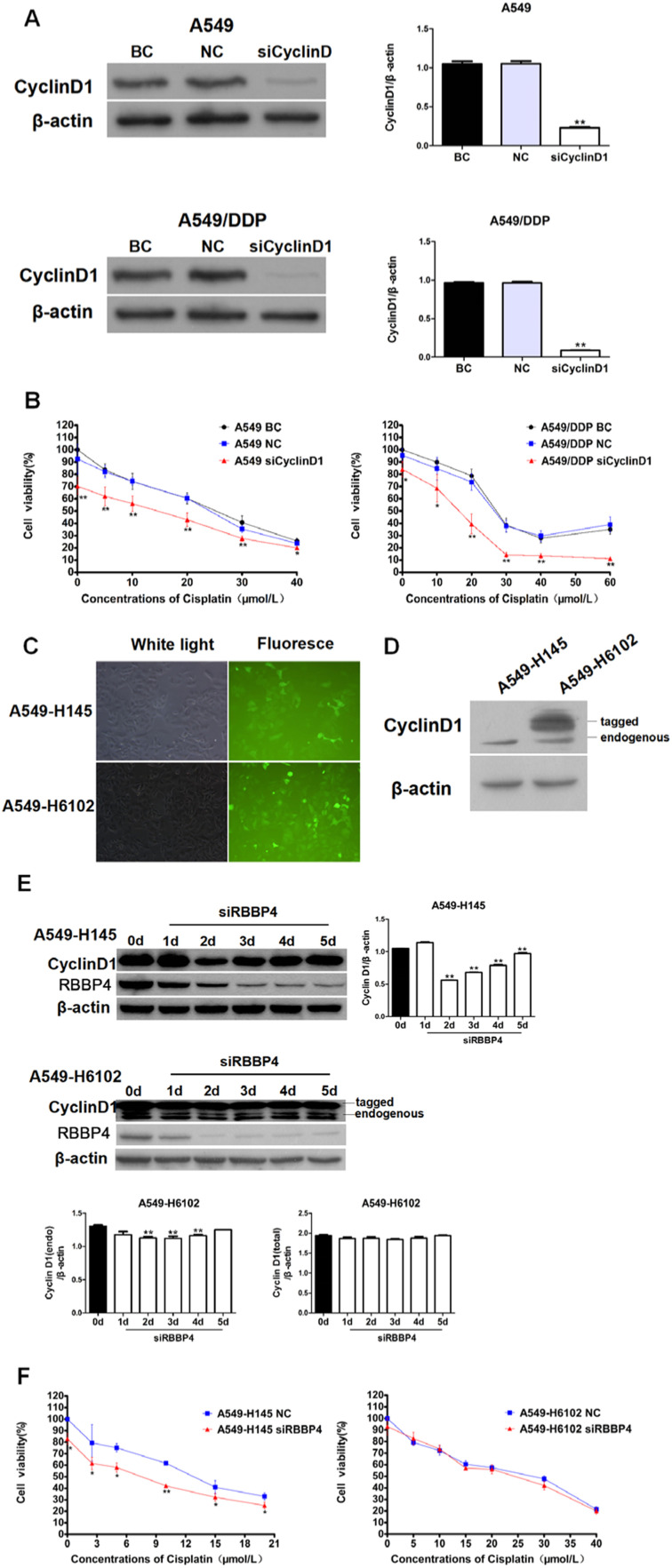
**Cyclin D1 suppression was crucial for cisplatin sensitization by RBBP4 downregulation.** (A) siRNA was used to downregulate cyclinD1 protein expression in A549 and A549/DDP cells. (B) Cells with siCyclinD1 and cisplatin combined treatment resulted in enhancements of cisplatin sensitivity. (C‒D) CyclinD1 overexpression could antagonize cisplatin toxicity. Scale bar is 200 μm. (E) CyclinD1 expression of the A549-H6102 cells maintained at a high level. (F) No changes in cisplatin sensitivity were found in the A549-H6102 cells after RBBP4 downregulation. Data are reported as means ± SD with three replicates. * *p* < 0.05, ** *p* < 0.01, *** *p* < 0.001. RBBP4, Retinoblastoma-Binding Protein-4; BC, Blank Control; NC, siRBBP4 Negative Control.

To validate cyclin D1′s role, the authors modulated its expression in A549 and A549/DDP cells. Cyclin D1 knockdown via siRNA significantly enhanced cisplatin sensitivity, with CI values < 1 (0.676‒0.978 for A549 and 0.511‒0.910 for A549/DDP; Fig. 5A‒B). Conversely, cyclin D1 overexpression in A549-H6102 cells attenuated cisplatin sensitivity, even after RBBP4 downregulation (Fig. 5C‒F). These findings confirm that cyclin D1 overexpression antagonizes the sensitizing effects of RBBP4 downregulation, underscoring cyclin D1′s central role in mediating cisplatin sensitivity.

### Results replicated in HeLa cervical cancer cells

**Fig. 6:**
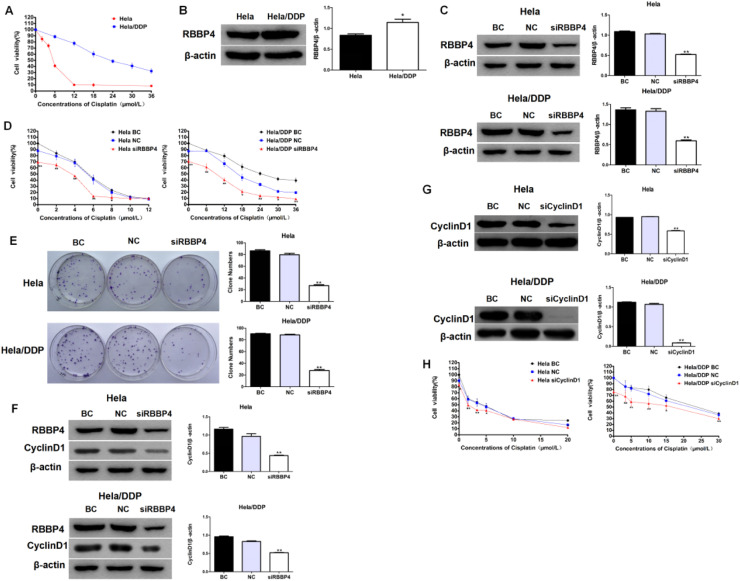
**Results replicated in HeLa cervical cancer cells.** (A) MTT showed the difference of cisplatin IC_50_ in HeLa/DDP cells. (B) RBBP4 expression in HeLa/DDP cells was higher than HeLa cells. (D) Cisplatin sensitivities in HeLa and HeLa/DDP cells were increase after RBBP4 downregulation. (E) CyclinD1 expression in HeLa and HeLa/DDP cells was inhibited after RBBP4 downregulation. (F‒H) Cisplatin sensitivities were significantly enhanced in HeLa and HeLa/DDP cells after RBBP4 downregulation. Data are reported as means ± SD with three replicates. * *p* < 0.05, ** *p* < 0.01, *** *p* < 0.001. RBBP4, Retinoblastoma-Binding Protein-4; BC, Blank Control; NC, siRBBP4 Negative Control.

Similar experiments in HeLa and cisplatin-resistant HeLa/DDP cells (IC_50_ = 4.76 ± 0.32 μM vs. 25.69 ± 4.1 μM, respectively; Fig. A) showed that RBBP4 expression was significantly higher in HeLa/DDP cells (Fig. 6B). RBBP4 knockdown enhanced cisplatin sensitivity in both cell types (CI values < 1, ranging 0.563‒0.956 for HeLa and 0.282‒0.595 for HeLa/DDP; Fig. 6C‒D). Cyclin D1 expression was suppressed following RBBP4 downregulation, and cyclin D1 knockdown further enhanced cisplatin sensitivity (CI values < 1, ranging 0.366‒0.850 for HeLa and 0.533‒0.867 for HeLa/DDP; Fig. 6E‒H). These results confirm that RBBP4 downregulation enhances cisplatin sensitivity in HeLa cells through cyclin D1 suppression, consistent with findings in A549 cells.

### RBBP4 was overexpressed in lung and cervical adenocarcinoma clinical specimens

**Fig. 7:**
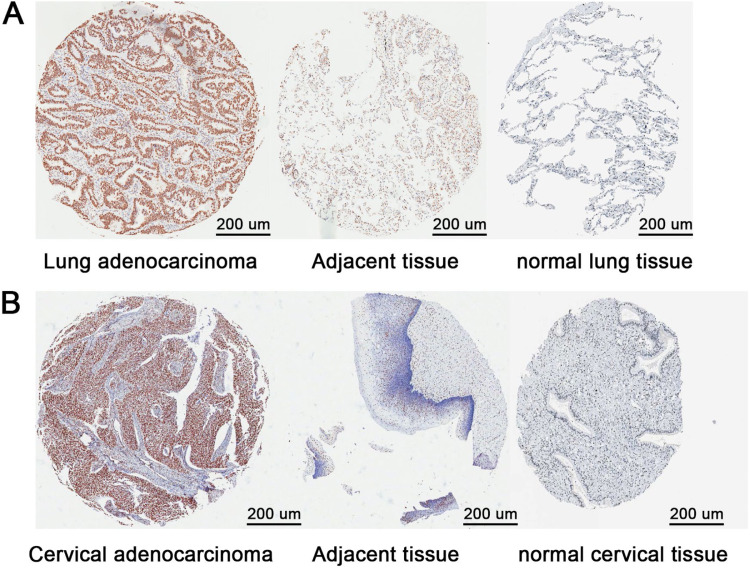
**RBBP4 was overexpressed in lung and cervical adenocarcinoma clinical specimens.** (A‒B) Tissue microarrays were used to study RBBP4 expression in clinical specimens. Scale bar is 200 μm.

In order to confirm the higher expression of RBBP4 at the tissue level compared to normal tissue, the authors used immunohistochemical results from the HPA database. Tissue microarray analysis of 75 lung adenocarcinoma and 11 cervical adenocarcinoma clinical specimens revealed significantly higher RBBP4 expression and positive cell proportions in tumors compared to adjacent normal tissues (Fig. 7A‒B; Table 1, Table 2, Table 3, Table 4). Similar trends were observed in lung and cervical squamous cell carcinoma specimens (data not shown). Despite limited sample sizes, these results suggest that RBBP4 overexpression may be a biomarker for cisplatin resistance and a potential target for improving chemotherapy efficacy.

**Table 1:** Comparison of RBBP4 expression between human lung adenocarcinoma and adjacent tissues.

Tissue	Total cases	RBBP4 expression		p-value
		Negative or weak n ( %)	Moderate n ( %)	
Cancer	75	55 (73.33 %)	20 (36.67 %)	0.000
Adjacent	75	75 (100 %)	0 (0.00 %)	

Chi-Square test.

**Table 2:** Distribution of the proportion of different RBBP4 expression levels in human lung adenocarcinoma and adjacent tissues.

Tissue	Total cases	Positive rate			p-value
		≤ 30 %	30 %‒70 %	≥ 70 %	
Cancer	75	1 (1.33 %)	0 (0.00 %)	74 (98.67 %)	0.000
Adjacent	75	48 (64.00 %)	26 (34.67 %)	1 (1.33 %)	

Chi-Square test.

**Table 3:** Comparison of RBBP4 expression between human cervical adenocarcinoma and adjacent tissues.

Tissue	Total cases	RBBP4 expression			p-value
		Negative or weak n ( %)	Moderate n ( %)	Strong n ( %)	
Cancer	11	2 (18.18 %)	2 (36.67 %)	7 (63.64 %)	0.000
Adjacent	16	14 (87.50 %)	2 (12.50 %)	0 (0.00 %)	

Chi-Square test.

**Table 4:** Distribution of the proportion of different RBBP4 expression levels in human cervical adenocarcinoma and adjacent tissues.

Tissue	Total cases	Positive rate			p-value
		≤ 30 %	30 %‒70 %	≥ 70 %	
Cancer	11	0 (0.00 %)	2 (18.18 %)	9 (81.82 %)	0.000
Adjacent	16	8 (50.00 %)	7 (43.75 %)	1 (6.25 %)	

Chi-Square test.

## Discussion

This study demonstrates that the expression of RBBP4 is closely associated with the application of cisplatin and is significantly upregulated in the cisplatin-resistant human cancer cell lines A549/DDP and HeLa/DDP. Functional experiments further confirmed that silencing RBBP4 markedly inhibits the expression of cyclin D1, thereby enhancing the effects of cisplatin on A549 and HeLa cells and reversing cisplatin resistance in A549/DDP and HeLa/DDP cells. These findings suggest that RBBP4 plays a critical role in mediating tumor cell resistance to cisplatin and could serve as a potential target for enhancing cisplatin sensitivity.

Previous studies have shown that the RBBP4 protein is overexpressed in various cancers, including hepatocellular carcinoma, ^28^ colorectal cancer, ^29^ thyroid cancer, ^30^ cervical cancer, ^31^^,^^32^ and acute myeloid leukemia.^33^^,^^34^ However, its biological roles in tumor cells remain unclear. The previous study has shown that RBBP downregulation inhibits epithelial-mesenchymal transition of human cervical cancer MS751 cells.^27^ This study further reveals that RBBP4 can regulate cisplatin sensitivity by modulating cyclin D1 expression, consistent with previous findings that RBBP4 knockdown inhibits epithelial-mesenchymal transition in cervical cancer cells. Although the data in this study focused on lung adenocarcinoma and cervical adenocarcinoma, the observed overexpression of RBBP4 in clinical specimens of gastric cancer, colorectal cancer, and esophageal cancer (data not shown) suggests that RBBP4 may similarly contribute to cisplatin resistance in these cancers. If this hypothesis holds true, RBBP4 could play a pivotal role in guiding clinical drug selection and monitoring therapeutic efficacy.

Notably, the role of RBBP4 may vary across different tumor types. For example, Kitange et al. reported that RBBP4 knockdown in glioblastoma enhanced sensitivity to temozolomide by suppressing the expression of MGMT, RAD51, and FIGNL1 genes.^26^ However, in contrast to the present findings, RBBP4 knockdown did not alter cyclin D1 expression in their study. Further analysis suggests that this discrepancy may stem from the histological and developmental differences between glioblastoma and lung or cervical adenocarcinoma. Glioblastoma originates from glial cells derived from the ectoderm, whereas lung and cervical adenocarcinomas arise from epithelial cells of mesodermal and endodermal origin, respectively.^35^^,^^36^ The different tissue origins may account for the distinct gene regulatory networks influenced by RBBP4.^37^^,^^38^ In addition, the nervous system is derived from the ectoderm, while the uterus and lungs are derived from the mesoderm and endoderm, respectively, which are very different from each other. Recent research has shown that frequent genomic alterations in glioblastoma affect the epigenomic machinery. In glioblastoma, Receptor Tyrosine Kinases (RTKs) are commonly activated by somatic mutations or structural variations, which leads to Pyruvate Kinase M2 (PKM2) nuclear translocation. Nuclear PKM2 has been found to phosphorylate histone H3 at threonine 11, which causes dissociation of HDAC3 from the cyclinD1 and MYC gene promoters. Subsequent expressions of cyclin D1 and MYC, owing to increased acetylation of H3 at lysine 9, have been found to induce cell proliferation and gliomagenesis.^39^^,^^40^ This indicates that cyclinD1 expression in glioblastoma cells is specified, which might be the reason why cyclinD1 expression is unaffected after RBBP4 downregulation.

Beyond glioblastoma, the authors observed that RBBP4 knockdown significantly reduced cyclin D1 expression in hepatocellular carcinoma, colorectal cancer, and breast cancer cell lines (data not shown), further supporting the importance of RBBP4 in regulating gene expression across various tumors. The heterogeneous regulatory effects of RBBP4 suggest that its function is closely linked to the genomic and epigenetic contexts of specific cancers. However, further research is needed to elucidate the underlying mechanisms driving these differences.

In conclusion, this study provides new insights into the role of RBBP4 in regulating cisplatin sensitivity, particularly in lung adenocarcinoma and cervical adenocarcinoma models. The overexpression of RBBP4 appears to be a key factor contributing to cisplatin resistance in tumors, while its knockdown significantly enhances cisplatin sensitivity. These findings not only deepen our understanding of the mechanisms by which RBBP4 influences cancer drug resistance but also highlight its potential as a therapeutic target. Future studies should focus on exploring the regulatory networks and mechanisms of RBBP4 in various tumors to evaluate its clinical applicability in cancer therapy.

## Funding

This work was supported by grants from the Combination Project of Guangdong Province and the Ministry of Education (No. 2012B091100465), Sichuan Medical Association youth innovation research project (Q22070).

## Declaration of competing interest

The authors declare that the research was conducted in the absence of any commercial or financial relationships that could be construed as a potential conflict of interest.

## Appendix Supplementary materials

Image, application 1
