# Supplementary material for: RBBP4 downregulation increases the sensitivity of A549 and HeLa cells to cisplatin by inhibiting cyclin D1 expression
Source: Clinics (Sao Paulo). 2025 Apr 4;80:100637. doi: 10.1016/j.clinsp.2025.100637 (PMC12002758; doi:10.1016/j.clinsp.2025.100637)
Supplement: Supplementary file 1 [file mmc1.docx]

**CLINICS-D-24-00935_ Supplementary Material**

**Supplementary Table S1** List of genes elevated by siRBBP4 in A549 cells.

| **id** | **Count (siNT)** | **Count (siRBBP4)** | **foldChange** | **log2FoldChange** | **pval** | **padj** | **Description** |
| --- | --- | --- | --- | --- | --- | --- | --- |
| ENSG00000224231.1 | 0 | 16,07966 | Inf | Inf | 0.0001 | 0.010093 | Ensg00000224231.1 |
| VTCN1 | 0 | 6,934241 | Inf | Inf | 0.000449 | 0.028739 | V-set domain containing T cell activation inhibitor 1 |
| DUX2 | 0 | 6,48228 | Inf | Inf | 0.000767 | 0.041135 | Double homeobox 2 |
| FAM19A2 | 0 | 16,30872 | Inf | Inf | 0.001064 | 0.049751 | Family with sequence similarity 19 (chemokine (C-C motif)-like), member A2 |
| KRT6A | 5,36672 | 252,1904 | 46,99152 | 5,554329 | 3.49E-10 | 8.24E-07 | Keratin 6A |
| CLCA2 | 0,307874 | 8,49923 | 27,60617 | 4,786919 | 0.000664 | 0.037444 | Chloride channel accessory 2 |
| GPR87 | 33,1627 | 631,2253 | 19,0342 | 4,250522 | 1.06E-07 | 5.51E-05 | G protein-coupled receptor 87 |
| ENSG00000235167.1 | 0,624173 | 10,03785 | 16,08185 | 4,007361 | 0.00099 | 0.047244 | Ensg00000235167.1 |
| SFTPD | 1,153502 | 16,16177 | 14,01105 | 3,808494 | 2.83E-05 | 0.00411 | Surfactant protein D |
| EDN3 | 1,777674 | 23,99419 | 13,49752 | 3,754622 | 2.27E-06 | 0.000581 | Endothelin 3 |
| ENSG00000205562.1 | 3,641769 | 41,80551 | 11,47945 | 3,520982 | 1.19E-07 | 6.00E-05 | Ensg00000205562.1 |
| DIO2 | 151,7489 | 1625,923 | 10,71456 | 3,421501 | 1.04E-06 | 0.000336 | Deiodinase, iodothyronine, type II |
| SERPINB5 | 1,691254 | 17,03799 | 10,07417 | 3,332589 | 4.49E-05 | 0.005646 | Serpin peptidase inhibitor, clade B (ovalbumin), member 5 |
| DAND5 | 1,691254 | 16,95372 | 10,02435 | 3,325436 | 4.55E-05 | 0.005701 | DAN domain family member 5, BMP antagonist |
| SCEL | 48,6059 | 479,5204 | 9,865479 | 3,302389 | 5.95E-09 | 6.77E-06 | Sciellin |
| ALPP | 3,127358 | 30,32033 | 9,695191 | 3,277269 | 1.34E-06 | 0.000399 | Alkaline phosphatase, placental |
| ENSG00000224294.1 | 5,358296 | 50,23924 | 9,375973 | 3,228968 | 8.41E-08 | 4.94E-05 | Ensg00000224294.1 |
| TP63 | 57,89801 | 528,7968 | 9,133247 | 3,191128 | 3.49E-07 | 0.000132 | Tumor protein p63 |
| GPR1 | 6,545494 | 59,19932 | 9,044286 | 3,177007 | 3.31E-08 | 2.67E-05 | G protein-coupled receptor 1 |
| UGT2B11 | 1,68283 | 15,0731 | 8,956994 | 3,163015 | 0.000242 | 0.019097 | UDP glucuronosyltransferase 2 family, polypeptide B11 |
| PVRL4 | 49,06141 | 419,9665 | 8,560016 | 3,097614 | 2.33E-16 | 3.58E-12 | Poliovirus receptor-related 4 |
| INPP5D | 3,563773 | 30,19118 | 8,47169 | 3,08265 | 6.54E-06 | 0.001286 | Inositol polyphosphate-5-phosphatase, 145kda |
| ENSG00000249279.1 | 8,462312 | 71,01162 | 8,391515 | 3,068931 | 3.74E-08 | 2.94E-05 | Ensg00000249279.1 |
| CIDEC | 14,08225 | 117,9623 | 8,376668 | 3,066376 | 2.40E-07 | 0.000102 | Cell death-inducing DFFA-like effector c |
| ARHGAP25 | 5,615625 | 44,17254 | 7,866006 | 2,975631 | 0.00025 | 0.019385 | Rho gtpase activating protein 25 |
| DHRS9 | 12,61245 | 90,01683 | 7,137139 | 2,835346 | 7.78E-06 | 0.001456 | Dehydrogenase/reductase (SDR family) member 9 |
| PLAC1 | 15,04175 | 106,7017 | 7,093704 | 2,826539 | 8.96E-09 | 9.47E-06 | Placenta-specific 1 |
| TRIML2 | 181,2772 | 1281,734 | 7,070574 | 2,821827 | 1.48E-19 | 4.55E-15 | Tripartite motif family-like 2 |
| ENSG00000224347.2 | 4,527586 | 31,90423 | 7,04663 | 2,816934 | 5.17E-06 | 0.001087 | Ensg00000224347.2 |
| DIO2-AS1 | 3,572197 | 25,10512 | 7,027921 | 2,813098 | 0.000306 | 0.022228 | DIO2 antisense RNA 1 |
| ENSG00000258766.1 | 3,572197 | 25,10512 | 7,027921 | 2,813098 | 0.000306 | 0.022228 | Ensg00000258766.1 |
| ENSG00000226965.1 | 7,435421 | 51,3291 | 6,903322 | 2,787291 | 1.39E-06 | 0.000409 | Ensg00000226965.1 |
| MAP2 | 139,6855 | 949,8586 | 6,799982 | 2,765531 | 2.69E-05 | 0.004011 | Microtubule-associated protein 2 |
| MKRN9P | 9,662497 | 65,20812 | 6,748578 | 2,754584 | 9.63E-08 | 5.27E-05 | Makorin ring finger protein 9, pseudogene |
| SLC52A3 | 8,90329 | 59,66712 | 6,701693 | 2,744526 | 0.000232 | 0.018618 | Solute carrier family 52, riboflavin transporter, member 3 |
| GDF6 | 6,819672 | 43,16014 | 6,328771 | 2,661925 | 7.33E-06 | 0.001397 | Growth differentiation factor 6 |
| C1orf116 | 441,1874 | 2755,711 | 6,246123 | 2,642961 | 0.000887 | 0.044668 | Chromosome 1 open reading frame 116 |
| PPL | 284,4147 | 1727,353 | 6,073361 | 2,602495 | 0.000471 | 0.029539 | Periplakin |
| ENSG00000233840.1 | 4,392552 | 26,59862 | 6,055391 | 2,59822 | 0.000304 | 0.022228 | Ensg00000233840.1 |
| TRIM29 | 53,16725 | 321,8456 | 6,053455 | 2,597759 | 0.000182 | 0.015422 | Tripartite motif containing 29 |
| RLN2 | 2,922752 | 17,58699 | 6,017271 | 2,589109 | 0.00069 | 0.03814 | Relaxin 2 |
| ENSG00000251095.2 | 8,641398 | 51,83873 | 5,998882 | 2,584694 | 8.49E-06 | 0.001551 | Ensg00000251095.2 |
| ENSG00000251584.1 | 2,528458 | 14,65909 | 5,79764 | 2,535466 | 0.000925 | 0.045852 | Ensg00000251584.1 |
| MRO | 14,25053 | 81,95603 | 5,751086 | 2,523834 | 2.07E-06 | 0.000538 | Maestro |
| ENSG00000234527.1 | 5,657746 | 31,93657 | 5,644752 | 2,49691 | 7.83E-05 | 0.008462 | Ensg00000234527.1 |
| UG0898H09 | 7,43953 | 41,6901 | 5,603862 | 2,486421 | 0.000379 | 0.025825 | Uncharacterized LOC643763 |
| ENSG00000255546.1 | 3,530076 | 19,66079 | 5,569508 | 2,47755 | 0.000688 | 0.03814 | Ensg00000255546.1 |
| ENSG00000223586.1 | 10,57313 | 53,88094 | 5,096024 | 2,349372 | 2.89E-05 | 0.004166 | Ensg00000223586.1 |
| ENSG00000250697.1 | 33,4619 | 170,3207 | 5,08999 | 2,347663 | 2.03E-08 | 1.83E-05 | Ensg00000250697.1 |
| ENSG00000228842.2 | 7,015854 | 35,55445 | 5,06773 | 2,34134 | 8.01E-05 | 0.008588 | Ensg00000228842.2 |
| PCDH9-AS2 | 7,015854 | 35,55445 | 5,06773 | 2,34134 | 8.01E-05 | 0.008588 | PCDH9 antisense RNA 2 |
| STAT4 | 164,3788 | 831,772 | 5,060094 | 2,339164 | 9.46E-16 | 7.25E-12 | Signal transducer and activator of transcription 4 |
| ENSG00000231858.1 | 16,3789 | 81,92793 | 5,002041 | 2,322517 | 1.10E-06 | 0.000353 | Ensg00000231858.1 |
| KPNA7 | 10,49321 | 51,37079 | 4,895623 | 2,291492 | 1.84E-05 | 0.002939 | Karyopherin alpha 7 (importin alpha 8) |
| ENSG00000259705.1 | 3,483847 | 17,03679 | 4,890224 | 2,289901 | 0.000449 | 0.028739 | Ensg00000259705.1 |
| EPCAM | 22,06217 | 106,0321 | 4,806062 | 2,264855 | 3.41E-05 | 0.00463 | Epithelial cell adhesion molecule |
| CAPN8 | 10,31798 | 48,44288 | 4,694996 | 2,231124 | 0.000106 | 0.010591 | Calpain 8 |
| ENSG00000271894.1 | 6,904161 | 31,73397 | 4,596354 | 2,20049 | 0.000193 | 0.01619 | Ensg00000271894.1 |
| MGC34034 | 12,55541 | 57,42015 | 4,573338 | 2,193247 | 5.05E-05 | 0.006125 | Uncharacterized protein MGC34034 |
| AOX1 | 360,9644 | 1635,072 | 4,52973 | 2,179425 | 1.15E-07 | 5.90E-05 | Aldehyde oxidase 1 |
| ENSG00000259345.1 | 9,598965 | 41,60568 | 4,334393 | 2,11583 | 0.000279 | 0.020738 | Ensg00000259345.1 |
| DAW1 | 33,44891 | 140,1816 | 4,190915 | 2,067265 | 2.32E-06 | 0.000584 | Dynein assembly factor with WDR repeat domains 1 |
| PCDH9-AS3 | 10,03994 | 41,72948 | 4,156346 | 2,055316 | 0.000142 | 0.013099 | PCDH9 antisense RNA 3 |
| ENSG00000233554.1 | 56,3558 | 227,1678 | 4,030957 | 2,011122 | 8.75E-07 | 0.000295 | Ensg00000233554.1 |
| ENSG00000225263.1 | 9,41577 | 37,37516 | 3,969421 | 1,988929 | 0.000267 | 0.020256 | Ensg00000225263.1 |
| ENSG00000268621.1 | 500,9395 | 1982,334 | 3,957233 | 1,984492 | 2.52E-15 | 1.44E-11 | Ensg00000268621.1 |
| KMO | 503,1756 | 1979,394 | 3,933804 | 1,975925 | 1.56E-07 | 7.35E-05 | Kynurenine 3-monooxygenase (kynurenine 3-hydroxylase) |
| HAS2 | 234,7979 | 915,4353 | 3,898823 | 1,963039 | 0.000147 | 0.013235 | Hyaluronan synthase 2 |
| ID4 | 38,76842 | 150,5503 | 3,883322 | 1,957291 | 6.21E-05 | 0.00711 | Inhibitor of DNA binding 4, dominant negative helix-loop-helix protein |
| SERPINB7 | 44,23964 | 171,5909 | 3,878668 | 1,955561 | 4.13E-05 | 0.005353 | Serpin peptidase inhibitor, clade B (ovalbumin), member 7 |
| SEMA3E | 60,84533 | 235,9982 | 3,878658 | 1,955558 | 8.73E-05 | 0.009143 | Sema domain, immunoglobulin domain (Ig), short basic domain, secreted, (semaphorin) 3E |
| FGFBP1 | 167,6704 | 644,3685 | 3,843066 | 1,942258 | 0.00095 | 0.0462 | Fibroblast growth factor binding protein 1 |
| ACTBL2 | 9,624238 | 36,88262 | 3,832264 | 1,938197 | 0.00102 | 0.048305 | Actin, beta-like 2 |
| RBMS3 | 156,1679 | 597,7124 | 3,82737 | 1,936353 | 8.59E-10 | 1.65E-06 | RNA binding motif, single stranded interacting protein 3 |
| FDXR | 1833,769 | 7015,594 | 3,825779 | 1,935753 | 1.60E-06 | 0.000446 | Ferredoxin reductase |
| GREM2 | 23,60996 | 89,47094 | 3,789542 | 1,922023 | 2.53E-05 | 0.003877 | Gremlin 2, DAN family BMP antagonist |
| DOK7 | 8,372031 | 31,56969 | 3,770852 | 1,914891 | 0.000612 | 0.035576 | Docking protein 7 |
| FLRT2 | 92,82758 | 346,2334 | 3,729855 | 1,89912 | 7.07E-06 | 0.001364 | Fibronectin leucine rich transmembrane protein 2 |
| PROC | 46,7072 | 174,2039 | 3,729701 | 1,89906 | 2.16E-06 | 0.000556 | Protein C (inactivator of coagulation factors Va and viiia) |
| STON2 | 480,2509 | 1787,448 | 3,721905 | 1,896041 | 1.90E-13 | 7.28E-10 | Stonin 2 |
| HES2 | 76,99966 | 284,3167 | 3,69244 | 1,884575 | 0.000386 | 0.026066 | Hairy and enhancer of split 2 (Drosophila) |
| ENSG00000248279.1 | 14,68308 | 53,35189 | 3,633562 | 1,861384 | 0.000523 | 0.031493 | Ensg00000248279.1 |
| LOC340113 | 14,68308 | 53,35189 | 3,633562 | 1,861384 | 0.000523 | 0.031493 | Uncharacterized LOC340113 |
| CGB8 | 32,19407 | 116,8313 | 3,62897 | 1,85956 | 3.76E-05 | 0.004973 | Chorionic gonadotropin, beta polypeptide 8 |
| TPM4 | 5501,624 | 19884,93 | 3,614374 | 1,853746 | 3.69E-16 | 3.77E-12 | Tropomyosin 4 |
| BBC3 | 311,005 | 1117,537 | 3,59331 | 1,845313 | 4.30E-06 | 0.000949 | BCL2 binding component 3 |
| CEACAM1 | 32,67524 | 115,4331 | 3,532738 | 1,820787 | 2.57E-05 | 0.003891 | Carcinoembryonic antigen-related cell adhesion molecule 1 (biliary glycoprotein) |
| SMPDL3B | 47,74662 | 167,9906 | 3,518377 | 1,81491 | 0.000932 | 0.045977 | Sphingomyelin phosphodiesterase, acid-like 3B |
| AKAP6 | 60,99767 | 212,4201 | 3,482429 | 1,800094 | 3.31E-06 | 0.000781 | A kinase (PRKA) anchor protein 6 |
| ENSG00000233251.3 | 18,43918 | 64,20543 | 3,482011 | 1,799921 | 0.000169 | 0.014668 | Ensg00000233251.3 |
| PTGER4 | 104,9569 | 363,6071 | 3,464346 | 1,792583 | 2.54E-07 | 0.000105 | Prostaglandin E receptor 4 (subtype EP4) |
| OPN3 | 1008,287 | 3469,717 | 3,441201 | 1,782912 | 6.75E-09 | 7.40E-06 | Opsin 3 |
| B4GALT1 | 2130,436 | 7331,097 | 3,441126 | 1,782881 | 3.90E-14 | 1.71E-10 | UDP-Gal:betaglcnac beta 1,4- galactosyltransferase, polypeptide 1 |
| FLRT3 | 387,5678 | 1332,675 | 3,438561 | 1,781805 | 2.45E-07 | 0.000103 | Fibronectin leucine rich transmembrane protein 3 |
| ZNF608 | 182,1354 | 625,7905 | 3,435854 | 1,780669 | 0.000246 | 0.019329 | Zinc finger protein 608 |
| ENSG00000267922.1 | 15,0143 | 51,40059 | 3,423443 | 1,775448 | 0.000611 | 0.035567 | Ensg00000267922.1 |
| LINC00704 | 24,95097 | 84,99202 | 3,406361 | 1,768231 | 0.000105 | 0.010567 | Long intergenic non-protein coding RNA 704 |
| ENSG00000253878.1 | 25,18928 | 85,69343 | 3,40198 | 1,766375 | 0.000167 | 0.014545 | Ensg00000253878.1 |
| ENSG00000231298.2 | 29,75467 | 101,0775 | 3,397031 | 1,764274 | 7.56E-05 | 0.008343 | Ensg00000231298.2 |
| LOC100996255 | 69,2135 | 232,8741 | 3,364576 | 1,750425 | 0.00097 | 0.04669 | Uncharacterized LOC100996255 |
| COL4A4 | 471,0531 | 1578,758 | 3,351549 | 1,744828 | 2.57E-11 | 8.77E-08 | Collagen, type IV, alpha 4 |
| SERPINI1 | 34,66402 | 115,7354 | 3,338777 | 1,73932 | 3.76E-05 | 0.004973 | Serpin peptidase inhibitor, clade I (neuroserpin), member 1 |
| MPZL3 | 15,85993 | 52,74849 | 3,325897 | 1,733744 | 0.000637 | 0.036548 | Myelin protein zero-like 3 |
| AGMO | 35,23887 | 116,1016 | 3,294703 | 1,720148 | 0.000166 | 0.01453 | Alkylglycerol monooxygenase |
| ENSG00000223989.1 | 19,60953 | 64,08508 | 3,268058 | 1,708434 | 0.000318 | 0.022922 | Ensg00000223989.1 |
| ENSG00000256325.1 | 17,79359 | 57,65271 | 3,240082 | 1,69603 | 0.000976 | 0.046859 | Ensg00000256325.1 |
| CDC42BPG | 79,21975 | 255,2536 | 3,222096 | 1,688 | 5.00E-06 | 0.001059 | CDC42 binding protein kinase gamma (DMPK-like) |
| ENSG00000250337.1 | 812,0291 | 2607,396 | 3,210964 | 1,683006 | 4.04E-05 | 0.005273 | Ensg00000250337.1 |
| POF1B | 369,2541 | 1180,337 | 3,196545 | 1,676514 | 4.35E-10 | 9.52E-07 | Premature ovarian failure, 1B |
| PALMD | 74,71814 | 237,3431 | 3,176513 | 1,667444 | 0.00052 | 0.031433 | Palmdelphin |
| ARID5B | 211,0574 | 668,2336 | 3,166123 | 1,662717 | 1.84E-08 | 1.77E-05 | AT rich interactive domain 5B (MRF1-like) |
| ENSG00000229021.2 | 35,65047 | 112,2725 | 3,149258 | 1,655012 | 0.000138 | 0.012892 | Ensg00000229021.2 |
| MUC16 | 16,84059 | 52,98291 | 3,146143 | 1,653584 | 0.000753 | 0.040596 | Mucin 16, cell surface associated |
| ACTA2 | 285,6499 | 897,5225 | 3,142037 | 1,6517 | 2.06E-05 | 0.003223 | Actin, alpha 2, smooth muscle, aorta |
| NTN4 | 2543,115 | 7958,462 | 3,129415 | 1,645893 | 0.000937 | 0.045977 | Netrin 4 |
| GALNT5 | 219,0632 | 683,7669 | 3,121323 | 1,642158 | 5.68E-08 | 3.87E-05 | UDP-N-acetyl-alpha-D-galactosamine: polypeptide N-acetylgalactosaminyltransferase 5 (galnac-T5) |
| PTPRB | 46,60277 | 144,5183 | 3,101066 | 1,632764 | 0.000248 | 0.019329 | Protein tyrosine phosphatase, receptor type, B |
| SDC4 | 1507,17 | 4649,718 | 3,085065 | 1,625301 | 8.53E-08 | 4.94E-05 | Syndecan 4 |
| CCDC68 | 224,3775 | 692,1875 | 3,084925 | 1,625235 | 2.86E-07 | 0.000117 | Coiled-coil domain containing 68 |
| MICAL2 | 1148,793 | 3515,46 | 3,060133 | 1,613594 | 0.000378 | 0.025825 | Microtubule associated monooxygenase, calponin and LIM domain containing 2 |
| ENSG00000248161.1 | 33,62414 | 101,1147 | 3,007204 | 1,588423 | 0.00028 | 0.020762 | Ensg00000248161.1 |
| MAML2 | 168,1702 | 504,998 | 3,002899 | 1,586356 | 5.17E-05 | 0.006214 | Mastermind-like 2 (Drosophila) |
| ENSG00000227954.2 | 111,4694 | 333,9854 | 2,996206 | 1,583137 | 3.60E-06 | 0.000835 | Ensg00000227954.2 |
| LOC643401 | 364,7019 | 1088,302 | 2,984085 | 1,577289 | 0.000234 | 0.018637 | Uncharacterized LOC643401 |
| COL15A1 | 23,66244 | 70,11339 | 2,963067 | 1,567091 | 0.000967 | 0.046637 | Collagen, type XV, alpha 1 |
| ALOX5AP | 50,01294 | 147,6755 | 2,952746 | 1,562057 | 0.000321 | 0.02307 | Arachidonate 5-lipoxygenase-activating protein |
| FGD4 | 507,2089 | 1484,567 | 2,926934 | 1,54939 | 1.17E-09 | 1.89E-06 | FYVE, rhogef and PH domain containing 4 |
| BTG2 | 417,4832 | 1219,855 | 2,921926 | 1,546919 | 1.20E-08 | 1.22E-05 | BTG family, member 2 |
| ENSG00000229425.1 | 29,16419 | 84,89579 | 2,91096 | 1,541495 | 0.000608 | 0.035504 | Ensg00000229425.1 |
| FHDC1 | 32,63049 | 94,96323 | 2,910261 | 1,541148 | 0.000863 | 0.044049 | FH2 domain containing 1 |
| NTN1 | 78,71011 | 228,552 | 2,903719 | 1,537902 | 1.57E-05 | 0.002555 | Netrin 1 |
| RHOB | 4691,076 | 13572,43 | 2,893244 | 1,532688 | 1.14E-06 | 0.000357 | Ras homolog family member B |
| ENSG00000247903.1 | 246,0397 | 710,5221 | 2,887835 | 1,529988 | 1.35E-07 | 6.57E-05 | Ensg00000247903.1 |
| GPNMB | 41,76198 | 120,481 | 2,884944 | 1,528543 | 0.000306 | 0.022228 | Glycoprotein (transmembrane) nmb |
| GPR115 | 346,4756 | 992,1126 | 2,863442 | 1,51775 | 7.12E-08 | 4.46E-05 | G protein-coupled receptor 115 |
| MMP24 | 1523,86 | 4360,01 | 2,861161 | 1,516601 | 0.000999 | 0.047507 | Matrix metallopeptidase 24 (membrane-inserted) |
| HSF2BP | 84,12531 | 240,2885 | 2,856317 | 1,514156 | 0.000129 | 0.012227 | Heat shock transcription factor 2 binding protein |
| LOC553103 | 167,469 | 474,9056 | 2,835782 | 1,503747 | 5.87E-06 | 0.001197 | Uncharacterized LOC553103 |
| MGC16121 | 97,09787 | 274,9581 | 2,831762 | 1,5017 | 3.71E-05 | 0.004973 | Mgc16121 |
| PERP | 4366,179 | 12353,47 | 2,829354 | 1,500473 | 3.73E-11 | 1.14E-07 | PERP, TP53 apoptosis effector |
| PTK2B | 253,4676 | 716,7382 | 2,827731 | 1,499645 | 0.000844 | 0.043322 | Protein tyrosine kinase 2 beta |
| ENSG00000258451.1 | 38,20856 | 106,9498 | 2,799107 | 1,484967 | 0.000692 | 0.03816 | Ensg00000258451.1 |
| ENSG00000245904.2 | 193,1145 | 539,0868 | 2,791539 | 1,481061 | 8.68E-06 | 0.001576 | Ensg00000245904.2 |
| TP53TG1 | 158,2226 | 441,3168 | 2,789214 | 1,479859 | 3.42E-06 | 0.000802 | TP53 target 1 (non-protein coding) |
| STYK1 | 36,79625 | 102,304 | 2,780284 | 1,475232 | 0.000268 | 0.020256 | Serine/threonine/tyrosine kinase 1 |
| HABP4 | 311,969 | 866,1537 | 2,77641 | 1,473221 | 1.85E-07 | 8.48E-05 | Hyaluronan binding protein 4 |
| VAMP8 | 235,1466 | 652,0885 | 2,773115 | 1,471507 | 0.000122 | 0.01176 | Vesicle-associated membrane protein 8 |
| TTC9 | 168,1177 | 465,9322 | 2,771465 | 1,470649 | 2.45E-06 | 0.000603 | Tetratricopeptide repeat domain 9 |
| LAMB3 | 1700,335 | 4690,05 | 2,758309 | 1,463784 | 5.36E-05 | 0.006353 | Laminin, beta 3 |
| CCDC85A | 35,82812 | 98,48779 | 2,748896 | 1,458852 | 0.000603 | 0.035364 | Coiled-coil domain containing 85A |
| ENSG00000259171.1 | 209,9104 | 571,7496 | 2,723779 | 1,44561 | 1.29E-06 | 0.000389 | Ensg00000259171.1 |
| ENSG00000230615.1 | 84,01046 | 228,6373 | 2,721533 | 1,44442 | 0.000146 | 0.013235 | Ensg00000230615.1 |
| ENSG00000182165.12 | 169,9875 | 461,6663 | 2,715884 | 1,441422 | 4.15E-06 | 0.00093 | Ensg00000182165.12 |
| MED11 | 257,8335 | 696,8736 | 2,702805 | 1,434457 | 3.32E-07 | 0.000129 | Mediator complex subunit 11 |
| TLE4 | 531,9332 | 1435,129 | 2,69795 | 1,431864 | 0.000358 | 0.02492 | Transducin-like enhancer of split 4 (E(sp1) homolog, Drosophila) |
| KIAA1324 | 54,56966 | 146,9628 | 2,693123 | 1,42928 | 0.000783 | 0.041367 | Kiaa1324 |
| STOM | 3110,715 | 8348,742 | 2,683866 | 1,424312 | 4.99E-07 | 0.00018 | Stomatin |
| SNAI2 | 145,1571 | 388,4924 | 2,676357 | 1,420271 | 2.62E-05 | 0.00394 | Snail family zinc finger 2 |
| RNASE4 | 221,6688 | 592,7454 | 2,674014 | 1,419007 | 1.62E-06 | 0.000447 | Ribonuclease, rnase A family, 4 |
| UPK3B | 126,1691 | 336,9159 | 2,670352 | 1,41703 | 2.67E-05 | 0.003995 | Uroplakin 3B |
| FKBP5 | 1354,132 | 3595,283 | 2,655047 | 1,408737 | 2.86E-08 | 2.37E-05 | FK506 binding protein 5 |
| ENSG00000272168.1 | 161,3362 | 428,2535 | 2,654416 | 1,408394 | 1.31E-05 | 0.002245 | Ensg00000272168.1 |
| WNT9A | 53,58756 | 141,5605 | 2,641668 | 1,401449 | 0.000947 | 0.046181 | Wingless-type MMTV integration site family, member 9A |
| GRIN2C | 46,89664 | 123,8343 | 2,640579 | 1,400854 | 0.000744 | 0.040198 | Glutamate receptor, ionotropic, N-methyl D-aspartate 2C |
| ENSG00000261488.1 | 76,17014 | 201,0753 | 2,639818 | 1,400438 | 0.00027 | 0.020337 | Ensg00000261488.1 |
| MESDC2 | 1352,565 | 3566,799 | 2,637062 | 1,398932 | 4.15E-09 | 5.09E-06 | Mesoderm development candidate 2 |
| HYPK | 336,4302 | 886,2154 | 2,634173 | 1,39735 | 4.12E-06 | 0.00093 | Huntingtin interacting protein K |
| LPCAT1 | 1843,308 | 4853,231 | 2,632892 | 1,396648 | 1.77E-09 | 2.59E-06 | Lysophosphatidylcholine acyltransferase 1 |
| FAM212B | 153,138 | 402,4691 | 2,628146 | 1,394045 | 2.21E-05 | 0.003405 | Family with sequence similarity 212, member B |
| IL6R | 766,0784 | 2009,245 | 2,622767 | 1,391089 | 1.99E-08 | 1.83E-05 | Interleukin 6 receptor |
| HSDL2 | 1085,485 | 2843,726 | 2,619775 | 1,389443 | 5.35E-09 | 6.32E-06 | Hydroxysteroid dehydrogenase like 2 |
| COBL | 187,0224 | 488,9869 | 2,61459 | 1,386585 | 0.000142 | 0.013099 | Cordon-bleu WH2 repeat protein |
| GAREM | 295,9028 | 756,591 | 2,556891 | 1,35439 | 4.22E-06 | 0.000939 | GRB2 associated, regulator of MAPK1 |
| SNX10 | 179,9938 | 458,5205 | 2,547424 | 1,349039 | 0.00013 | 0.012274 | Sorting nexin 10 |
| SULT2B1 | 218,4946 | 555,6493 | 2,543081 | 1,346577 | 0.000159 | 0.014002 | Sulfotransferase family, cytosolic, 2B, member 1 |
| EFNB2 | 237,8055 | 604,0667 | 2,540172 | 1,344926 | 0.000437 | 0.028493 | Ephrin-B2 |
| LINC00294 | 515,6419 | 1306,236 | 2,533223 | 1,340974 | 6.04E-07 | 0.000208 | Long intergenic non-protein coding RNA 294 |
| IL1R1 | 900,4776 | 2279,14 | 2,531034 | 1,339727 | 5.80E-08 | 3.87E-05 | Interleukin 1 receptor, type I |
| ENSG00000261898.2 | 50,62553 | 127,8826 | 2,52605 | 1,336883 | 0.00068 | 0.038038 | Ensg00000261898.2 |
| ENSG00000233006.2 | 312,5501 | 789,0065 | 2,524416 | 1,33595 | 5.89E-06 | 0.001197 | Ensg00000233006.2 |
| TSC22D2 | 641,4905 | 1615,665 | 2,51861 | 1,332628 | 4.19E-07 | 0.000155 | TSC22 domain family, member 2 |
| CCDC92 | 185,153 | 465,9542 | 2,51659 | 1,33147 | 2.74E-05 | 0.00402 | Coiled-coil domain containing 92 |
| ENSG00000223749.3 | 248,7158 | 624,7083 | 2,511735 | 1,328684 | 8.08E-06 | 0.001503 | Ensg00000223749.3 |
| IL15 | 198,2303 | 497,0981 | 2,50768 | 1,326353 | 4.77E-05 | 0.005903 | Interleukin 15 |
| FAS | 1011,437 | 2535,339 | 2,506671 | 1,325773 | 4.80E-08 | 3.51E-05 | Fas cell surface death receptor |
| KIAA1217 | 117,5463 | 293,8724 | 2,500057 | 1,321961 | 0.000188 | 0.015817 | Kiaa1217 |
| THSD7A | 710,8424 | 1771,803 | 2,49254 | 1,317617 | 2.73E-06 | 0.000664 | Thrombospondin, type I, domain containing 7A |
| KLF6 | 1106,293 | 2755,465 | 2,49072 | 1,316563 | 0.00051 | 0.031126 | Kruppel-like factor 6 |
| MGLL | 2170,495 | 5392,974 | 2,484674 | 1,313057 | 4.14E-05 | 0.005353 | Monoglyceride lipase |
| TMEM117 | 72,78402 | 180,5695 | 2,480895 | 1,310861 | 0.000663 | 0.037444 | Transmembrane protein 117 |
| CBLB | 433,7299 | 1075,348 | 2,479303 | 1,309935 | 0.000343 | 0.024199 | Cbl proto-oncogene, E3 ubiquitin protein ligase B |
| MISP | 2978,714 | 7350,031 | 2,467519 | 1,303061 | 3.69E-09 | 4.72E-06 | Mitotic spindle positioning |
| F3 | 84,71333 | 208,0267 | 2,455655 | 1,296108 | 0.000466 | 0.029327 | Coagulation factor III (thromboplastin, tissue factor) |
| FAM210B | 388,2496 | 953,0335 | 2,454692 | 1,295542 | 0.000115 | 0.011304 | Family with sequence similarity 210, member B |
| ENSG00000233766.2 | 368,1495 | 900,5194 | 2,44607 | 1,290466 | 0.000564 | 0.03323 | Ensg00000233766.2 |
| ELFN2 | 533,8825 | 1302,486 | 2,43965 | 1,286674 | 0.000464 | 0.029286 | Extracellular leucine-rich repeat and fibronectin type III domain containing 2 |
| ENSG00000224818.1 | 112,8413 | 275,2883 | 2,439606 | 1,286648 | 0.000301 | 0.022095 | Ensg00000224818.1 |
| KITLG | 812,7756 | 1979,336 | 2,435279 | 1,284087 | 1.65E-07 | 7.69E-05 | KIT ligand |
| GPR64 | 311,1939 | 757,0207 | 2,432634 | 1,282519 | 0.000108 | 0.01077 | G protein-coupled receptor 64 |
| SULF2 | 908,3402 | 2206,107 | 2,428723 | 1,280198 | 0.000824 | 0.042609 | Sulfatase 2 |
| THSD1 | 409,6746 | 987,5261 | 2,410514 | 1,269341 | 2.05E-05 | 0.003223 | Thrombospondin, type I, domain containing 1 |
| GANAB | 3488,435 | 8408,559 | 2,41041 | 1,269279 | 5.65E-08 | 3.87E-05 | Glucosidase, alpha; neutral AB |
| ENSG00000203721.1 | 109,537 | 263,5149 | 2,405716 | 1,266467 | 0.000449 | 0.028739 | Ensg00000203721.1 |
| ARHGEF37 | 93,43774 | 223,8326 | 2,395526 | 1,260343 | 0.000533 | 0.031899 | Rho guanine nucleotide exchange factor (GEF) 37 |
| ENSG00000266401.1 | 71,67071 | 170,8087 | 2,383243 | 1,252926 | 0.000957 | 0.046287 | Ensg00000266401.1 |
| KLHL4 | 469,6089 | 1116,575 | 2,377671 | 1,249549 | 0.000146 | 0.013235 | Kelch-like family member 4 |
| PPP1R3C | 527,4826 | 1244,416 | 2,359161 | 1,238274 | 5.97E-05 | 0.006929 | Protein phosphatase 1, regulatory subunit 3C |
| G3BP2 | 3218,598 | 7589,019 | 2,357865 | 1,237481 | 1.05E-07 | 5.51E-05 | Gtpase activating protein (SH3 domain) binding protein 2 |
| THRB | 211,2385 | 497,2572 | 2,354008 | 1,235119 | 0.000117 | 0.011459 | Thyroid hormone receptor, beta |
| WIPF1 | 213,4155 | 502,1174 | 2,352769 | 1,23436 | 9.44E-05 | 0.00962 | WAS/WASL interacting protein family, member 1 |
| ASTN2 | 388,8621 | 912,5535 | 2,346728 | 1,230651 | 1.27E-05 | 0.002202 | Astrotactin 2 |
| GJD3 | 410,2687 | 955,3212 | 2,328526 | 1,219417 | 1.94E-06 | 0.000518 | Gap junction protein, delta 3, 31.9kda |
| SYNPO2 | 370,2037 | 857,2876 | 2,315719 | 1,21146 | 1.20E-05 | 0.002111 | Synaptopodin 2 |
| ENSG00000126005.10 | 595,8965 | 1374,305 | 2,306281 | 1,205568 | 2.46E-06 | 0.000603 | Ensg00000126005.10 |
| ANXA4 | 2752,728 | 6338,131 | 2,302491 | 1,203195 | 1.59E-06 | 0.000446 | Annexin A4 |
| APH1B | 691,6803 | 1592,417 | 2,302244 | 1,20304 | 3.31E-05 | 0.004589 | APH1B gamma secretase subunit |
| TM7SF3 | 3161,68 | 7272,271 | 2,300129 | 1,201714 | 7.12E-05 | 0.007939 | Transmembrane 7 superfamily member 3 |
| PEMT | 428,6118 | 985,0113 | 2,298143 | 1,200469 | 2.94E-05 | 0.004211 | Phosphatidylethanolamine N-methyltransferase |
| JARID2 | 953,0244 | 2187,101 | 2,294906 | 1,198435 | 9.14E-07 | 0.000305 | Jumonji, AT rich interactive domain 2 |
| NR3C1 | 6930,027 | 15891,89 | 2,293193 | 1,197358 | 1.01E-07 | 5.43E-05 | Nuclear receptor subfamily 3, group C, member 1 (glucocorticoid receptor) |
| LRRN2 | 124,3068 | 284,7938 | 2,291056 | 1,196013 | 0.000913 | 0.045548 | Leucine rich repeat neuronal 2 |
| ENSG00000238266.1 | 250,7859 | 574,2629 | 2,289853 | 1,195255 | 7.69E-05 | 0.00842 | Ensg00000238266.1 |
| LINC00707 | 250,7859 | 574,2629 | 2,289853 | 1,195255 | 7.69E-05 | 0.00842 | Long intergenic non-protein coding RNA 707 |
| CYFIP2 | 1664,916 | 3796,741 | 2,28044 | 1,189312 | 3.70E-07 | 0.000138 | Cytoplasmic FMR1 interacting protein 2 |
| MMP24-AS1 | 511,0992 | 1163,942 | 2,277331 | 1,187344 | 6.40E-06 | 0.001274 | MMP24 antisense RNA 1 |
| C7orf55 | 224,3993 | 509,3026 | 2,269626 | 1,182455 | 0.000122 | 0.011811 | Chromosome 7 open reading frame 55 |
| PDGFC | 289,9525 | 656,2998 | 2,263474 | 1,178538 | 4.40E-05 | 0.00558 | Platelet derived growth factor C |
| SETD7 | 2496,193 | 5643,76 | 2,260947 | 1,176927 | 8.62E-05 | 0.009086 | SET domain containing (lysine methyltransferase) 7 |
| TCP11L1 | 1036,551 | 2343,209 | 2,260581 | 1,176694 | 1.49E-06 | 0.000426 | T-complex 11, testis-specific-like 1 |
| SDPR | 3095,197 | 6994,697 | 2,259855 | 1,17623 | 0.000159 | 0.014002 | Serum deprivation response |
| ATP2B4 | 1295,066 | 2926,07 | 2,259398 | 1,175938 | 0.000443 | 0.028739 | Atpase, Ca++ transporting, plasma membrane 4 |
| CFH | 254,7693 | 575,5851 | 2,259241 | 1,175838 | 2.03E-05 | 0.003206 | Complement factor H |
| TSC22D3 | 681,0212 | 1533,152 | 2,251254 | 1,170729 | 0.000213 | 0.017457 | TSC22 domain family, member 3 |
| ENSG00000258714.2 | 135,5089 | 304,3677 | 2,246109 | 1,167428 | 0.000704 | 0.038379 | Ensg00000258714.2 |
| PLA2G12A | 275,2196 | 617,2779 | 2,242856 | 1,165337 | 7.81E-05 | 0.008462 | Phospholipase A2, group XIIA |
| HECW1 | 339,4007 | 759,9246 | 2,239019 | 1,162867 | 3.35E-05 | 0.00463 | HECT, C2 and WW domain containing E3 ubiquitin protein ligase 1 |
| ATL1 | 250,8033 | 561,0809 | 2,237135 | 1,161653 | 0.000195 | 0.016284 | Atlastin gtpase 1 |
| ALS2CL | 329,2096 | 734,1743 | 2,230112 | 1,157116 | 4.74E-05 | 0.005903 | ALS2 C-terminal like |
| OGFR | 927,5818 | 2066,37 | 2,227696 | 1,155552 | 9.59E-07 | 0.000316 | Opioid growth factor receptor |
| ELF3 | 2203,782 | 4887,345 | 2,217708 | 1,14907 | 5.24E-05 | 0.00623 | E74-like factor 3 (ets domain transcription factor, epithelial-specific ) |
| PKP2 | 237,4433 | 526,4284 | 2,21707 | 1,148654 | 9.32E-05 | 0.009538 | Plakophilin 2 |
| ITGB4 | 2342,818 | 5186,447 | 2,213764 | 1,146501 | 1.24E-06 | 0.000379 | Integrin, beta 4 |
| CDH4 | 444,2187 | 978,8222 | 2,203469 | 1,139777 | 4.83E-05 | 0.005949 | Cadherin 4, type 1, R-cadherin (retinal) |
| DDB2 | 2022,696 | 4440,658 | 2,195415 | 1,134494 | 3.44E-07 | 0.000132 | Damage-specific DNA binding protein 2, 48kda |
| ATXN1 | 432,9669 | 946,4801 | 2,186034 | 1,128316 | 5.22E-05 | 0.00623 | Ataxin 1 |
| TCTA | 327,3689 | 714,728 | 2,183249 | 1,126477 | 0.000152 | 0.013688 | T-cell leukemia translocation altered |
| LINC00673 | 945,1154 | 2061,792 | 2,181524 | 1,125337 | 0.000118 | 0.011517 | Long intergenic non-protein coding RNA 673 |
| CHST14 | 232,4792 | 506,0819 | 2,176891 | 1,122269 | 0.000374 | 0.025666 | Carbohydrate (N-acetylgalactosamine 4‒0) sulfotransferase 14 |
| CTTN | 5795,12 | 12614,25 | 2,176702 | 1,122144 | 8.14E-07 | 0.000277 | Cortactin |
| ENSG00000249007.1 | 730,9551 | 1587,043 | 2,171191 | 1,118487 | 0.000267 | 0.020256 | Ensg00000249007.1 |
| ANG | 161,6304 | 350,5872 | 2,169067 | 1,117075 | 0.000545 | 0.032268 | Angiogenin, ribonuclease, rnase A family, 5 |
| LRRC37A | 255,0842 | 551,6561 | 2,162643 | 1,112796 | 0.000428 | 0.028205 | Leucine rich repeat containing 37A |
| CDRT1 | 235,9348 | 510,0181 | 2,161691 | 1,11216 | 0.000355 | 0.024832 | CMT1A duplicated region transcript 1 |
| KIF3C | 2722,878 | 5885,999 | 2,161683 | 1,112155 | 6.63E-06 | 0.001295 | Kinesin family member 3C |
| WBP4 | 265,1005 | 570,5324 | 2,152136 | 1,105769 | 0.000369 | 0.025507 | WW domain binding protein 4 |
| BTG1 | 1840,333 | 3942,928 | 2,142508 | 1,099301 | 1.12E-05 | 0.001983 | B-cell translocation gene 1, anti-proliferative |
| UFC1 | 1045,812 | 2237,047 | 2,139052 | 1,096971 | 2.42E-06 | 0.000603 | Ubiquitin-fold modifier conjugating enzyme 1 |
| MDM2 | 6312,209 | 13479,41 | 2,135451 | 1,094541 | 1.60E-06 | 0.000446 | MDM2 oncogene, E3 ubiquitin protein ligase |
| TMEM164 | 3096,203 | 6603,671 | 2,132829 | 1,092768 | 2.29E-06 | 0.000581 | Transmembrane protein 164 |
| KIF1C | 4190,952 | 8903,271 | 2,124403 | 1,087057 | 4.31E-07 | 0.000157 | Kinesin family member 1C |
| CDC42EP3 | 2249,42 | 4778,152 | 2,12417 | 1,086899 | 3.86E-06 | 0.000884 | CDC42 effector protein (Rho gtpase binding) 3 |
| LMO7 | 1676,543 | 3556,565 | 2,121368 | 1,084995 | 3.18E-06 | 0.000756 | LIM domain 7 |
| MFN1 | 989,8721 | 2098,504 | 2,119975 | 1,084047 | 1.40E-05 | 0.002369 | Mitofusin 1 |
| DLG5 | 1702,469 | 3601,913 | 2,1157 | 1,081135 | 4.78E-06 | 0.00102 | Discs, large homolog 5 (Drosophila) |
| ENSG00000227036.2 | 1185,693 | 2508,369 | 2,11553 | 1,081019 | 0.000159 | 0.014002 | Ensg00000227036.2 |
| ANKRA2 | 309,4619 | 654,613 | 2,115326 | 1,08088 | 0.000221 | 0.017885 | Ankyrin repeat, family A (RFXANK-like), 2 |
| TMEM56 | 1229,49 | 2591,995 | 2,108187 | 1,076003 | 8.96E-06 | 0.001607 | Transmembrane protein 56 |
| EPS8L2 | 2226,487 | 4675,468 | 2,099931 | 1,070342 | 8.14E-06 | 0.001505 | EPS8-like 2 |
| EXD2 | 946,0642 | 1986,041 | 2,099267 | 1,069886 | 1.25E-05 | 0.002174 | Exonuclease 3'-5' domain containing 2 |
| COL4A3 | 214,5579 | 450,3521 | 2,098977 | 1,069686 | 0.000489 | 0.030206 | Collagen, type IV, alpha 3 (Goodpasture antigen) |
| ABTB2 | 813,957 | 1702,575 | 2,091726 | 1,064694 | 0.000238 | 0.018881 | Ankyrin repeat and BTB (POZ) domain containing 2 |
| ENSG00000257181.1 | 832,4462 | 1740,447 | 2,090763 | 1,064029 | 2.14E-05 | 0.003335 | Ensg00000257181.1 |
| EMC6 | 441,3888 | 922,7729 | 2,090612 | 1,063925 | 4.23E-05 | 0.005457 | ER membrane protein complex subunit 6 |
| TTPAL | 920,746 | 1924,731 | 2,090404 | 1,063781 | 3.38E-05 | 0.00463 | Tocopherol (alpha) transfer protein-like |
| USP53 | 1066,008 | 2225,907 | 2,088078 | 1,062175 | 2.57E-05 | 0.003891 | Ubiquitin specific peptidase 53 |
| SLC16A4 | 2020,039 | 4214,397 | 2,086295 | 1,060943 | 3.08E-06 | 0.000739 | Solute carrier family 16, member 4 (monocarboxylic acid transporter 5) |
| PRKAG2 | 396,5471 | 826,4909 | 2,084219 | 1,059507 | 0.000226 | 0.018223 | Protein kinase, AMP-activated, gamma 2 non-catalytic subunit |
| SESN2 | 604,8284 | 1257,469 | 2,079051 | 1,055925 | 0.000153 | 0.013691 | Sestrin 2 |
| RAC1 | 3029,758 | 6286,064 | 2,074774 | 1,052954 | 6.44E-06 | 0.001275 | Ras-related C3 botulinum toxin substrate 1 (rho family, small GTP binding protein Rac1) |
| PRSS23 | 13976,94 | 28993,06 | 2,07435 | 1,052659 | 7.40E-06 | 0.0014 | Protease, serine, 23 |
| EEPD1 | 920,2083 | 1906,712 | 2,072044 | 1,051055 | 8.19E-05 | 0.008724 | Endonuclease/exonuclease/phosphatase family domain containing 1 |
| EMP1 | 355,1728 | 735,5384 | 2,070931 | 1,05028 | 0.000181 | 0.01539 | Epithelial membrane protein 1 |
| BCL7B | 671,1206 | 1388,944 | 2,069589 | 1,049344 | 4.48E-05 | 0.005646 | B-cell CLL/lymphoma 7B |
| ZNF219 | 835,7638 | 1723,901 | 2,062665 | 1,044509 | 1.44E-05 | 0.002373 | Zinc finger protein 219 |
| TPM2 | 7941,453 | 16379,69 | 2,062556 | 1,044433 | 4.79E-06 | 0.00102 | Tropomyosin 2 (beta) |
| ZDHHC16 | 815,8235 | 1681,057 | 2,060565 | 1,04304 | 3.02E-05 | 0.004261 | Zinc finger, DHHC-type containing 16 |
| SERPINE1 | 20815,35 | 42862,77 | 2,059191 | 1,042077 | 0.000415 | 0.02756 | Serpin peptidase inhibitor, clade E (nexin, plasminogen activator inhibitor type 1), member 1 |
| EPN1 | 1365,076 | 2804,797 | 2,054683 | 1,038916 | 1.44E-05 | 0.002373 | Epsin 1 |
| HSPB8 | 333,0168 | 682,837 | 2,050458 | 1,035946 | 0.000619 | 0.035753 | Heat shock 22kda protein 8 |
| MAP7 | 1087,928 | 2223,48 | 2,043776 | 1,031237 | 1.77E-05 | 0.002865 | Microtubule-associated protein 7 |
| EIF3F | 2861,825 | 5837,029 | 2,039618 | 1,028299 | 7.06E-06 | 0.001364 | Eukaryotic translation initiation factor 3, subunit F |
| MARCKS | 3290,889 | 6707,82 | 2,038301 | 1,027367 | 5.29E-06 | 0.001096 | Myristoylated alanine-rich protein kinase C substrate |
| EDA2R | 1049,32 | 2134,486 | 2,034162 | 1,024435 | 1.29E-05 | 0.002221 | Ectodysplasin A2 receptor |
| DSG2 | 2569,208 | 5225,524 | 2,033905 | 1,024252 | 6.03E-06 | 0.001216 | Desmoglein 2 |
| ZDHHC18 | 719,0038 | 1460,939 | 2,031893 | 1,022825 | 4.76E-05 | 0.005903 | Zinc finger, DHHC-type containing 18 |
| SVEP1 | 982,5889 | 1995,244 | 2,030599 | 1,021905 | 3.18E-05 | 0.004454 | Sushi, von Willebrand factor type A, EGF and pentraxin domain containing 1 |
| SESN1 | 653,4862 | 1326,822 | 2,030375 | 1,021746 | 9.09E-05 | 0.00939 | Sestrin 1 |
| ZNF512 | 1013,961 | 2053,711 | 2,025434 | 1,018231 | 0.000389 | 0.026163 | Zinc finger protein 512 |
| STX12 | 374,3012 | 758,0807 | 2,025323 | 1,018152 | 0.00034 | 0.024111 | Syntaxin 12 |
| ANK2 | 268,8179 | 544,2168 | 2,024481 | 1,017552 | 0.000254 | 0.019541 | Ankyrin 2, neuronal |
| C14orf93 | 217,969 | 438,5004 | 2,011756 | 1,008455 | 0.000681 | 0.038038 | Chromosome 14 open reading frame 93 |
| VPS53 | 989,5312 | 1988,13 | 2,009164 | 1,006595 | 0.000111 | 0.010941 | Vacuolar protein sorting 53 homolog (S. Cerevisiae) |
| MSRB3 | 1261,376 | 2527,156 | 2,003492 | 1,002517 | 1.34E-05 | 0.002279 | Methionine sulfoxide reductase B3 |

**Supplementary Table S2** List of genes supressioned by siRBBP4 in A549 cells.

| **id** | **Count (siNT)** | **Count (siRBBP4)** | **foldChange** | **log2FoldChange** | **pval** | **padj** | **Description** |
| --- | --- | --- | --- | --- | --- | --- | --- |
| PTK7 | 646,7294 | 323,3263 | 0.499941 | -1.00017 | 0.000341 | 0.024134 | Protein tyrosine kinase 7 |
| TOP1 | 2162,296 | 1077,472 | 0.4983 | -1.00491 | 4.29E-05 | 0.005489 | Topoisomerase (DNA) I |
| ENTPD7 | 1982,646 | 984,0167 | 0.496315 | -1.01067 | 2.02E-05 | 0.003205 | Ectonucleoside triphosphate diphosphohydrolase 7 |
| FBXO41 | 636,8228 | 315,9151 | 0.49608 | -1.01136 | 0.000391 | 0.026253 | F-box protein 41 |
| ARHGEF40 | 924,4336 | 458,0528 | 0.495496 | -1.01306 | 0.000188 | 0.015803 | Rho guanine nucleotide exchange factor (GEF) 40 |
| MAPK8IP1 | 502,1422 | 248,1691 | 0.494221 | -1.01677 | 0.001054 | 0.049647 | Mitogen-activated protein kinase 8 interacting protein 1 |
| ENSG00000261609.2 | 1181,177 | 581,2948 | 0.492132 | -1.02288 | 6.01E-05 | 0.006929 | Ensg00000261609.2 |
| DBT | 502,7251 | 247,0957 | 0.491513 | -1.0247 | 0.000682 | 0.038038 | Dihydrolipoamide branched chain transacylase E2 |
| CDC25C | 530,4172 | 260,6249 | 0.491358 | -1.02515 | 0.001055 | 0.049647 | Cell division cycle 25C |
| TSN | 3446,852 | 1693,547 | 0.491331 | -1.02523 | 2.80E-05 | 0.004091 | Translin |
| CDC20 | 4074,431 | 1989,015 | 0.48817 | -1.03454 | 0.000357 | 0.024891 | Cell division cycle 20 |
| KIF26A | 444,4858 | 216,5315 | 0.48715 | -1.03756 | 0.000687 | 0.03814 | Kinesin family member 26A |
| WARS | 3571,424 | 1739,755 | 0.487132 | -1.03762 | 4.38E-06 | 0.000954 | Tryptophanyl-trna synthetase |
| MED7 | 645,4336 | 310,9012 | 0.481693 | -1.05381 | 0.000355 | 0.024832 | Mediator complex subunit 7 |
| C17orf51 | 1780,416 | 857,337 | 0.481538 | -1.05428 | 1.41E-05 | 0.002369 | Chromosome 17 open reading frame 51 |
| RRM1 | 5135,19 | 2468,252 | 0.480654 | -1.05693 | 0.000771 | 0.041136 | Ribonucleotide reductase M1 |
| TNFAIP8L1 | 542,5572 | 260,7058 | 0.480513 | -1.05735 | 0.000486 | 0.030206 | Tumor necrosis factor, alpha-induced protein 8-like 1 |
| TMEM121 | 518,3305 | 248,6753 | 0.479762 | -1.05961 | 0.000436 | 0.028493 | Transmembrane protein 121 |
| RC3H2 | 2559,797 | 1227,989 | 0.479721 | -1.05973 | 1.13E-05 | 0.001986 | Ring finger and CCCH-type domains 2 |
| LOC401397 | 831,6207 | 397,6599 | 0.478175 | -1.06439 | 0.000145 | 0.013194 | Uncharacterized LOC401397 |
| TPRG1L | 624,1065 | 297,6819 | 0.476973 | -1.06802 | 0.000967 | 0.046637 | Tumor protein p63 regulated 1-like |
| HIST1H1D | 22543,1 | 10729,05 | 0.475935 | -1.07116 | 8.80E-06 | 0.001588 | Histone cluster 1, h1d |
| SUV420H2 | 418,3928 | 198,815 | 0.475187 | -1.07343 | 0.000499 | 0.030538 | Suppressor of variegation 4-20 homolog 2 (Drosophila) |
| SSR2 | 4248,22 | 2009,767 | 0.473085 | -1.07983 | 5.26E-06 | 0.001096 | Signal sequence receptor, beta (translocon-associated protein beta) |
| GNS | 7019,957 | 3310,519 | 0.471587 | -1.0844 | 1.44E-06 | 0.000418 | Glucosamine (N-acetyl)-6-sulfatase |
| PCYOX1L | 326,0591 | 152,9108 | 0.468966 | -1.09244 | 0.00101 | 0.04787 | Prenylcysteine oxidase 1 like |
| CCND1 | 30094,96 | 14112,08 | 0.468918 | -1.09259 | 1.42E-06 | 0.000416 | Cyclin D1 |
| C7orf60 | 794,0324 | 369,0314 | 0.464756 | -1.10545 | 4.29E-05 | 0.005489 | Chromosome 7 open reading frame 60 |
| SH2B3 | 859,0498 | 397,3944 | 0.462598 | -1.11217 | 3.52E-05 | 0.004733 | SH2B adaptor protein 3 |
| CCNE1 | 1218,729 | 563,6113 | 0.462458 | -1.1126 | 3.76E-05 | 0.004973 | Cyclin E1 |
| ORMDL1 | 1133,77 | 522,9797 | 0.461275 | -1.1163 | 2.74E-05 | 0.00402 | ORM1-like 1 (S. Cerevisiae) |
| LRRC41 | 2245,651 | 1033,841 | 0.460375 | -1.11912 | 4.46E-06 | 0.000964 | Leucine rich repeat containing 41 |
| NUF2 | 1085,45 | 495,9264 | 0.456885 | -1.1301 | 0.000476 | 0.029797 | NUF2, NDC80 kinetochore complex component, homolog (S. Cerevisiae) |
| ZNF697 | 415,7875 | 189,8464 | 0.456595 | -1.13101 | 0.000378 | 0.025825 | Zinc finger protein 697 |
| ENSG00000260822.1 | 457,5876 | 208,7052 | 0.456099 | -1.13258 | 0.000278 | 0.02072 | Ensg00000260822.1 |
| SCN1B | 574,4746 | 261,189 | 0.454657 | -1.13715 | 0.000153 | 0.013708 | Sodium channel, voltage-gated, type I, beta subunit |
| PIF1 | 560,1002 | 253,2753 | 0.452196 | -1.14498 | 0.000181 | 0.01539 | PIF1 5'-to-3' DNA helicase |
| HIST1H3J | 6907,062 | 3121,115 | 0.451873 | -1.14601 | 0.000657 | 0.037347 | Histone cluster 1, h3j |
| MAP2K6 | 778,9653 | 351,6533 | 0.451436 | -1.14741 | 3.80E-05 | 0.004998 | Mitogen-activated protein kinase kinase 6 |
| GNB4 | 2142,796 | 961,1217 | 0.448536 | -1.1567 | 4.11E-06 | 0.00093 | Guanine nucleotide binding protein (G protein), beta polypeptide 4 |
| SCAMP5 | 592,0828 | 264,9053 | 0.447413 | -1.16032 | 9.01E-05 | 0.009337 | Secretory carrier membrane protein 5 |
| ENSG00000242539.2 | 544,6747 | 241,6659 | 0.443688 | -1.17238 | 6.86E-05 | 0.007737 | Ensg00000242539.2 |
| AFAP1L1 | 616,0004 | 272,5469 | 0.442446 | -1.17643 | 5.03E-05 | 0.006125 | Actin filament associated protein 1-like 1 |
| BAI2 | 436,6529 | 193,0819 | 0.442186 | -1.17727 | 0.0007 | 0.038379 | Brain-specific angiogenesis inhibitor 2 |
| KBTBD6 | 946,4923 | 418,2735 | 0.44192 | -1.17814 | 1.41E-05 | 0.002369 | Kelch repeat and BTB (POZ) domain containing 6 |
| HAGHL | 437,0195 | 192,3934 | 0.44024 | -1.18364 | 0.000198 | 0.016451 | Hydroxyacylglutathione hydrolase-like |
| PSRC1 | 445,1996 | 195,9824 | 0.440212 | -1.18373 | 7.76E-05 | 0.008462 | Proline/serine-rich coiled-coil 1 |
| SPRED1 | 1535,173 | 674,6783 | 0.43948 | -1.18613 | 1.20E-06 | 0.000372 | Sprouty-related, EVH1 domain containing 1 |
| ENSG00000269728.1 | 644,2499 | 281,413 | 0.436807 | -1.19493 | 3.03E-05 | 0.004261 | Ensg00000269728.1 |
| ENSG00000271270.1 | 243,209 | 105,5571 | 0.434018 | -1.20417 | 0.000674 | 0.037817 | Ensg00000271270.1 |
| STMN3 | 251,7943 | 109,1968 | 0.433675 | -1.20532 | 0.000403 | 0.026878 | Stathmin-like 3 |
| GAS2L3 | 545,8791 | 236,176 | 0.432653 | -1.20872 | 6.34E-05 | 0.007227 | Growth arrest-specific 2 like 3 |
| GFPT2 | 3631,401 | 1570,67 | 0.432525 | -1.20915 | 2.99E-07 | 0.000119 | Glutamine-fructose-6-phosphate transaminase 2 |
| PPFIA3 | 351,4548 | 151,3574 | 0.43066 | -1.21538 | 0.000195 | 0.016275 | Protein tyrosine phosphatase, receptor type, f polypeptide (PTPRF), interacting protein (liprin), alpha 3 |
| DBNDD2 | 284,2839 | 122,3439 | 0.430358 | -1.21639 | 0.000875 | 0.04431 | Dysbindin (dystrobrevin binding protein 1) domain containing 2 |
| RIN1 | 894,9669 | 383,6717 | 0.428699 | -1.22196 | 4.33E-06 | 0.000949 | Ras and Rab interactor 1 |
| LIN52 | 322,3742 | 137,5345 | 0.42663 | -1.22894 | 0.000634 | 0.036398 | Lin-52 homolog (C. Elegans) |
| LOC100507032 | 194,8635 | 82,77195 | 0.424769 | -1.23525 | 0.000881 | 0.044543 | Uncharacterized LOC100507032 |
| OAF | 307,4332 | 130,5845 | 0.424757 | -1.23529 | 0.000273 | 0.020378 | OAF homolog (Drosophila) |
| ENSG00000272405.1 | 375,2064 | 159,1789 | 0.424244 | -1.23704 | 0.000245 | 0.019329 | Ensg00000272405.1 |
| LONRF1 | 373,8025 | 158,5253 | 0.424088 | -1.23756 | 8.81E-05 | 0.009195 | LON peptidase N-terminal domain and ring finger 1 |
| TGOLN2 | 6179,025 | 2611,407 | 0.422624 | -1.24255 | 3.98E-08 | 3.05E-05 | Trans-golgi network protein 2 |
| ENSG00000262292.2 | 213,6468 | 89,95482 | 0.421045 | -1.24796 | 0.000843 | 0.043322 | Ensg00000262292.2 |
| MALT1 | 2452,374 | 1021,138 | 0.416387 | -1.264 | 2.14E-07 | 9.51E-05 | Mucosa associated lymphoid tissue lymphoma translocation gene 1 |
| NFE2L3 | 2644,074 | 1088,876 | 0.411818 | -1.27992 | 5.75E-08 | 3.87E-05 | Nuclear factor (erythroid-derived 2)-like 3 |
| ENSG00000257151.1 | 384,6754 | 158,389 | 0.411747 | -1.28017 | 9.33E-05 | 0.009538 | Ensg00000257151.1 |
| NUDT11 | 191,8456 | 77,96372 | 0.406388 | -1.29907 | 0.000674 | 0.037817 | Nudix (nucleoside diphosphate linked moiety X)-type motif 11 |
| LRRC8B | 827,9284 | 335,2879 | 0.404972 | -1.30411 | 2.03E-06 | 0.000532 | Leucine rich repeat containing 8 family, member B |
| CACUL1 | 3623,616 | 1466,162 | 0.404613 | -1.30539 | 1.43E-08 | 1.42E-05 | CDK2-associated, cullin domain 1 |
| LRIG1 | 738,1979 | 296,0146 | 0.400996 | -1.31834 | 2.01E-06 | 0.000531 | Leucine-rich repeats and immunoglobulin-like domains 1 |
| E2F5 | 1091,482 | 436,6046 | 0.400011 | -1.32189 | 3.23E-07 | 0.000127 | E2F transcription factor 5, p130-binding |
| VWA1 | 703,177 | 279,0594 | 0.396855 | -1.33332 | 3.04E-06 | 0.000735 | Von Willebrand factor A domain containing 1 |
| 09/mar | 352,8753 | 139,1128 | 0.394226 | -1.3429 | 8.61E-05 | 0.009086 | Membrane-associated ring finger (C3HC4) 9 |
| IL27RA | 573,1515 | 225,8026 | 0.393967 | -1.34385 | 7.28E-06 | 0.001395 | Interleukin 27 receptor, alpha |
| LFNG | 527,4037 | 206,9525 | 0.392399 | -1.34961 | 1.53E-05 | 0.002516 | LFNG O-fucosylpeptide 3-beta-N-acetylglucosaminyltransferase |
| TTYH2 | 132,1177 | 51,4853 | 0.389693 | -1.35959 | 0.001061 | 0.049751 | Tweety homolog 2 (Drosophila) |
| TRAF1 | 661,2829 | 256,8104 | 0.388352 | -1.36456 | 0.000255 | 0.019592 | TNF receptor-associated factor 1 |
| PLEKHO1 | 348,9953 | 134,9297 | 0.386623 | -1.371 | 9.47E-05 | 0.00962 | Pleckstrin homology domain containing, family O member 1 |
| KBTBD8 | 168,9619 | 65,31785 | 0.386583 | -1.37115 | 0.000512 | 0.031169 | Kelch repeat and BTB (POZ) domain containing 8 |
| RHEBL1 | 156,1811 | 60,30929 | 0.38615 | -1.37277 | 0.000695 | 0.03828 | Ras homolog enriched in brain like 1 |
| NFKBIE | 1011,074 | 386,0341 | 0.381806 | -1.38909 | 2.94E-07 | 0.000119 | Nuclear factor of kappa light polypeptide gene enhancer in B-cells inhibitor, epsilon |
| MAP1A | 300,6143 | 113,8917 | 0.378863 | -1.40025 | 3.03E-05 | 0.004261 | Microtubule-associated protein 1A |
| MERTK | 238,6874 | 90,03856 | 0.377224 | -1.40651 | 0.000901 | 0.045256 | C-mer proto-oncogene tyrosine kinase |
| ENSG00000259932.1 | 3666,294 | 1375,258 | 0.375108 | -1.41462 | 5.46E-06 | 0.001124 | Ensg00000259932.1 |
| GALNT1 | 5075,054 | 1900,286 | 0.374437 | -1.41721 | 1.65E-09 | 2.53E-06 | UDP-N-acetyl-alpha-D-galactosamine:polypeptide N-acetylgalactosaminyltransferase 1 (galnac-T1) |
| ENSG00000267010.1 | 159,5554 | 59,73584 | 0.374389 | -1.41739 | 0.000547 | 0.032335 | Ensg00000267010.1 |
| ALKBH1 | 624,6086 | 233,572 | 0.373949 | -1.41909 | 1.64E-06 | 0.000448 | Alkb, alkylation repair homolog 1 (E. Coli) |
| LIN7C | 3369,844 | 1259,345 | 0.37371 | -1.42001 | 9.98E-10 | 1.70E-06 | Lin-7 homolog C (C. Elegans) |
| NRARP | 203,5345 | 75,12419 | 0.369098 | -1.43792 | 0.000128 | 0.012212 | NOTCH-regulated ankyrin repeat protein |
| ENSG00000260108.1 | 404,9215 | 148,8833 | 0.367684 | -1.44346 | 0.000178 | 0.015233 | Ensg00000260108.1 |
| ATP1A3 | 86,60803 | 31,77924 | 0.366932 | -1.44642 | 0.000911 | 0.045507 | Atpase, Na+/K+ transporting, alpha 3 polypeptide |
| ENSG00000264207.1 | 207,8572 | 75,68778 | 0.364134 | -1.45746 | 0.00017 | 0.014668 | Ensg00000264207.1 |
| PLXND1 | 1120,574 | 404,6576 | 0.361116 | -1.46946 | 1.02E-06 | 0.000334 | Plexin D1 |
| RMND5A | 4936,262 | 1772,286 | 0.359034 | -1.47781 | 8.56E-11 | 2.19E-07 | Required for meiotic nuclear division 5 homolog A (S. Cerevisiae) |
| ANKRD46 | 712,5939 | 255,6494 | 0.358759 | -1.47891 | 2.35E-07 | 0.000102 | Ankyrin repeat domain 46 |
| CLDN12 | 1938,782 | 689,1591 | 0.35546 | -1.49224 | 9.35E-10 | 1.69E-06 | Claudin 12 |
| C2CD4C | 109,7609 | 38,99087 | 0.355235 | -1.49316 | 0.000489 | 0.030206 | C2 calcium-dependent domain containing 4C |
| ENSG00000232803.1 | 88,26822 | 30,95958 | 0.350744 | -1.51151 | 0.00069 | 0.03814 | Ensg00000232803.1 |
| POM121L9P | 117,7253 | 41,23096 | 0.35023 | -1.51362 | 0.000464 | 0.029286 | POM121 transmembrane nucleoporin-like 9, pseudogene |
| LOC100335030 | 138,2423 | 48,22616 | 0.348852 | -1.51931 | 0.000398 | 0.026639 | FGFR1 oncogene partner 2 pseudogene |
| ANTXR2 | 306,4023 | 106,8856 | 0.348841 | -1.51936 | 0.000795 | 0.041709 | Anthrax toxin receptor 2 |
| RPS6KA5 | 398,2712 | 137,9318 | 0.346326 | -1.5298 | 1.12E-06 | 0.000355 | Ribosomal protein S6 kinase, 90kda, polypeptide 5 |
| MBOAT1 | 679,3483 | 233,5891 | 0.343843 | -1.54018 | 8.08E-08 | 4.86E-05 | Membrane bound O-acyltransferase domain containing 1 |
| SLCO4A1 | 714,3563 | 245,425 | 0.343561 | -1.54136 | 9.50E-08 | 5.27E-05 | Solute carrier organic anion transporter family, member 4A1 |
| NT5E | 5570,213 | 1895,429 | 0.340279 | -1.55521 | 2.60E-08 | 2.22E-05 | 5'-nucleotidase, ecto (CD73) |
| GATSL3 | 491,2061 | 165,1359 | 0.336185 | -1.57267 | 5.44E-07 | 0.000192 | GATS protein-like 3 |
| CCNH | 1172,585 | 392,9581 | 0.335121 | -1.57725 | 2.46E-09 | 3.43E-06 | Cyclin H |
| ACOX2 | 113,2606 | 37,88598 | 0.334503 | -1.57991 | 0.000199 | 0.01647 | Acyl-coa oxidase 2, branched chain |
| MED12L | 330,5947 | 110,502 | 0.334252 | -1.58099 | 3.62E-06 | 0.000835 | Mediator complex subunit 12-like |
| ENSG00000231864.2 | 610,6741 | 200,0708 | 0.327623 | -1.60989 | 1.49E-07 | 7.15E-05 | Ensg00000231864.2 |
| ENSG00000256196.1 | 70,46575 | 23,02811 | 0.326799 | -1.61353 | 0.000389 | 0.026163 | Ensg00000256196.1 |
| CACNA1G | 1229,817 | 400,781 | 0.325887 | -1.61756 | 4.67E-10 | 9.55E-07 | Calcium channel, voltage-dependent, T type, alpha 1G subunit |
| SAPCD2 | 2545,879 | 824,0478 | 0.323679 | -1.62736 | 5.97E-07 | 0.000208 | Suppressor APC domain containing 2 |
| RECK | 522,7653 | 167,4899 | 0.320392 | -1.64209 | 6.79E-08 | 4.43E-05 | Reversion-inducing-cysteine-rich protein with kazal motifs |
| KCNH3 | 548,4741 | 173,9742 | 0.317197 | -1.65655 | 1.07E-05 | 0.001915 | Potassium voltage-gated channel, subfamily H (eag-related), member 3 |
| ENSG00000259645.1 | 57,1282 | 17,80998 | 0.311755 | -1.68152 | 0.000779 | 0.041307 | Ensg00000259645.1 |
| ENSG00000267093.1 | 45,18615 | 13,84771 | 0.306459 | -1.70623 | 0.00095 | 0.0462 | Ensg00000267093.1 |
| HSPD1 | 30824,96 | 9386,013 | 0.304494 | -1.71551 | 1.34E-07 | 6.57E-05 | Heat shock 60kda protein 1 (chaperonin) |
| ALDOC | 238,3238 | 70,97846 | 0.297824 | -1.74747 | 6.15E-06 | 0.001233 | Aldolase C, fructose-bisphosphate |
| ADAM11 | 242,7297 | 70,97464 | 0.292402 | -1.77398 | 1.76E-06 | 0.000475 | ADAM metallopeptidase domain 11 |
| ZNF404 | 31,3759 | 8,751362 | 0.27892 | -1.84208 | 0.000978 | 0.046888 | Zinc finger protein 404 |
| RIMKLA | 331,0937 | 90,29037 | 0.272703 | -1.8746 | 6.97E-08 | 4.45E-05 | Ribosomal modification protein rimk-like family member A |
| TIGD3 | 56,08689 | 15,19211 | 0.270867 | -1.88434 | 0.000238 | 0.018875 | Tigger transposable element derived 3 |
| ABCG1 | 76,3692 | 20,48455 | 0.268231 | -1.89845 | 0.000108 | 0.010759 | ATP-binding cassette, sub-family G (WHITE), member 1 |
| ENSG00000229257.2 | 66,89158 | 17,77098 | 0.265668 | -1.9123 | 9.94E-05 | 0.010029 | Ensg00000229257.2 |
| SLC2A4 | 43,0019 | 11,37522 | 0.264528 | -1.91851 | 0.000446 | 0.028739 | Solute carrier family 2 (facilitated glucose transporter), member 4 |
| RAB37 | 41,76173 | 11,04561 | 0.264491 | -1.91871 | 0.000482 | 0.030107 | RAB37, member RAS oncogene family |
| ACBD7 | 142,9235 | 37,53001 | 0.262588 | -1.92913 | 8.43E-06 | 0.001548 | Acyl-coa binding domain containing 7 |
| DACT3 | 34,99503 | 9,140874 | 0.261205 | -1.93675 | 0.000702 | 0.038379 | Dishevelled-binding antagonist of beta-catenin 3 |
| PIK3IP1 | 244,1219 | 62,51554 | 0.256083 | -1.96531 | 2.27E-07 | 9.95E-05 | Phosphoinositide-3-kinase interacting protein 1 |
| HPCAL4 | 82,54908 | 21,03528 | 0.254822 | -1.97244 | 2.85E-05 | 0.004129 | Hippocalcin like 4 |
| B3GNT1 | 2140,413 | 532,7517 | 0.248901 | -2.00635 | 2.82E-15 | 1.44E-11 | UDP-glcnac:betagal beta-1,3-N-acetylglucosaminyltransferase 1 |
| PHOSPHO1 | 53,42771 | 13,25239 | 0.248043 | -2.01134 | 0.000145 | 0.013235 | Phosphatase, orphan 1 |
| ENSG00000250986.1 | 49,93351 | 12,31253 | 0.246579 | -2.01988 | 0.00014 | 0.012978 | Ensg00000250986.1 |
| PLA2G4C | 367,0156 | 90,412 | 0.246344 | -2.02126 | 4.20E-08 | 3.14E-05 | Phospholipase A2, group IVC (cytosolic, calcium-independent) |
| RPRML | 28,41752 | 6,912277 | 0.24324 | -2.03955 | 0.000655 | 0.037273 | Reprimo-like |
| SERPINB9 | 1244,67 | 301,6148 | 0.242325 | -2.04498 | 0.000453 | 0.02888 | Serpin peptidase inhibitor, clade B (ovalbumin), member 9 |
| SLC22A31 | 59,61092 | 14,3179 | 0.240189 | -2.05776 | 0.000143 | 0.013152 | Solute carrier family 22, member 31 |
| ENSG00000269420.1 | 42,87311 | 10,284 | 0.239871 | -2.05967 | 0.000175 | 0.01501 | Ensg00000269420.1 |
| CSF2 | 48,18665 | 10,51178 | 0.218147 | -2.19663 | 3.24E-05 | 0.004521 | Colony stimulating factor 2 (granulocyte-macrophage) |
| P2RY13 | 20,36635 | 4,309192 | 0.211584 | -2.2407 | 0.000935 | 0.045977 | Purinergic receptor P2Y, G-protein coupled, 13 |
| IL16 | 28,66014 | 6,059225 | 0.211416 | -2.24184 | 0.000374 | 0.025666 | Interleukin 16 |
| WNK3 | 68,87867 | 14,29513 | 0.207541 | -2.26853 | 7.73E-06 | 0.001455 | WNK lysine deficient protein kinase 3 |
| LOC728392 | 291,5082 | 58,99791 | 0.202389 | -2.3048 | 2.81E-09 | 3.75E-06 | Uncharacterized LOC728392 |
| CATSPER1 | 48,17437 | 9,683082 | 0.201001 | -2.31473 | 1.73E-05 | 0.002802 | Cation channel, sperm associated 1 |
| SOX8 | 54,23245 | 9,640351 | 0.17776 | -2.492 | 1.67E-06 | 0.000453 | SRY (sex determining region Y)-box 8 |
| KCNH2 | 146,5997 | 25,1501 | 0.171556 | -2.54325 | 8.02E-08 | 4.86E-05 | Potassium voltage-gated channel, subfamily H (eag-related), member 2 |
| COL13A1 | 52,28193 | 8,683078 | 0.166082 | -2.59003 | 1.26E-06 | 0.000383 | Collagen, type XIII, alpha 1 |
| GPR137C | 33,81601 | 5,559747 | 0.164412 | -2.60462 | 1.84E-05 | 0.002939 | G protein-coupled receptor 137C |
| P2RY14 | 29,29923 | 4,677936 | 0.159661 | -2.64692 | 5.21E-05 | 0.00623 | Purinergic receptor P2Y, G-protein coupled, 14 |
| SBSN | 25,61057 | 4,079024 | 0.159271 | -2.65044 | 0.000887 | 0.044668 | Suprabasin |
| ENSG00000205037.2 | 20,78592 | 2,961447 | 0.142474 | -2.81123 | 5.94E-05 | 0.006929 | Ensg00000205037.2 |
| TMEM86A | 46,70457 | 6,396818 | 0.136963 | -2.86814 | 5.34E-07 | 0.000191 | Transmembrane protein 86A |
| MMP9 | 72,56141 | 9,872528 | 0.136058 | -2.87771 | 9.24E-08 | 5.25E-05 | Matrix metallopeptidase 9 (gelatinase B, 92kda gelatinase, 92kda type IV collagenase) |
| SLC43A1 | 146,2042 | 18,64825 | 0.127549 | -2.97087 | 6.35E-11 | 1.77E-07 | Solute carrier family 43, member 1 |
| ITGAX | 43,62993 | 5,183012 | 0.118795 | -3.07346 | 1.97E-07 | 8.89E-05 | Integrin, alpha X (complement component 3 receptor 4 subunit) |
| ENSG00000272541.1 | 10,3329 | 1,144326 | 0.110746 | -3.17467 | 0.001 | 0.047507 | Ensg00000272541.1 |
| SNAR-D | 16,27518 | 1,729266 | 0.106252 | -3.23444 | 4.32E-05 | 0.005494 | Small ILF3/NF90-associated RNA D |
| PURG | 17,50281 | 1,750033 | 0.099986 | -3.32213 | 0.000669 | 0.037679 | Purine-rich element binding protein G |
| VWA5A | 27,36104 | 2,633041 | 0.096233 | -3.37732 | 0.000313 | 0.022695 | Von Willebrand factor A domain containing 5A |
| NKX2-3 | 13,59073 | 1,145523 | 0.084287 | -3.56855 | 5.51E-05 | 0.006503 | NK2 homeobox 3 |
| SNAR-G1 | 8,468806 | 0,582547 | 0.068787 | -3.86171 | 0.00091 | 0.045507 | Small ILF3/NF90-associated RNA G1 |
| OGDHL | 28,64547 | 1,44718 | 0.05052 | -4.30699 | 2.15E-08 | 1.89E-05 | Oxoglutarate dehydrogenase-like |
| ENSG00000251249.1 | 9,654073 | 0,302853 | 0.031371 | -4.99445 | 0.000649 | 0.037086 | Ensg00000251249.1 |
| KCNG4 | 5,759084 | 0 | 0 | #NOME? | 0.000921 | 0.045799 | Potassium voltage-gated channel, subfamily G, member 4 |
